# Fetal Pathogenesis of Scoliosis Suggested by Asymmetry of Gene Expression in Paravertebral Muscles

**DOI:** 10.1002/jsp2.70214

**Published:** 2026-08-16

**Authors:** Tian Cheng, Gamze Yazgeldi Gunaydin, Elisabet Einarsdottir, Sini Ezer, Juha Kere, Shintaro Katayama, Paul Gerdhem

**Affiliations:** ^1^ Department of Surgical Sciences, Orthopedics and Hand Surgery Uppsala University Uppsala Sweden; ^2^ Department of Orthopedics and Hand Surgery Uppsala University Hospital Uppsala Sweden; ^3^ Folkhälsan Research Centre Helsinki Finland; ^4^ Stem Cells and Metabolism Research Program University of Helsinki Helsinki Finland; ^5^ Department of Medicine, Huddinge Karolinska Institutet Stockholm Sweden; ^6^ National Genomics Infrastructure, Science for Life Laboratory (SciLifeLab) KTH Royal Institute of Technology Stockholm Sweden; ^7^ Department of Clinical Science, Intervention and Technology (CLINTEC) Karolinska Institutet Stockholm Sweden

**Keywords:** gene expression, genetics, scoliosis, spine

## Abstract

**Background:**

The dysfunction of paravertebral muscles may contribute to the development of idiopathic scoliosis. Several candidate genes have been linked to scoliosis, but the underlying mechanisms and cell types remain unclear.

**Methods:**

We initially included 40 idiopathic scoliosis cases and 19 controls. Muscle biopsies were obtained bilaterally in the cases, and at least unilaterally in the controls. RNA sequencing, differential gene expression analysis and gene set enrichment analysis were performed between cases and controls, and between convex and concave sides in the cases. Hounsfield units (HU) in paravertebral muscles from preoperative CT were assessed at biopsy sites in cases.

**Results:**

Qualified transcriptome analysis included 56 samples (30 convex, 26 concave) from 35 scoliosis cases and 22 samples from 17 controls. Among 14 212 expressed genes included in the downstream analysis, 22 differentially expressed genes were identified between cases and controls and 16 between convex and concave sides. Scoliosis cases showed decreased fetal muscle and immune cells. On the convex side, fibro‐adipogenic progenitors, endothelial cell types, satellite cells and myeloid cells were reduced, whereas fetal skeletal muscle cells were increased. Morphological asymmetry was verified by the HU findings.

**Conclusion:**

These results indicate asymmetric gene expression profiles between the convex and concave paraspinal muscles and support the hypothesis that idiopathic scoliosis may have a fetal developmental component, with the convex side exhibiting molecular signatures consistent with greater muscle mass.

## 1 Introduction

Idiopathic scoliosis is the most prevalent type of spinal deformity, accounting for 80% of all scoliosis cases. Its prevalence is approximately 2%–3%, and it is more common in females than in males [1, 2]. The progression can be rapid during a child's growth spurt, and if left untreated, a large curve can lead to respiratory dysfunction and ultimately premature death [3]. Brace treatment or surgery with spinal correction and fusion is indicated to halt progression [4, 5].

Idiopathic scoliosis is considered a polygenic and multifactorial disease, but its etiology and pathogenesis remain poorly understood [5]. Twin and family studies suggest a strong genetic component [6, 7, 8], although currently known single nucleotide variants explain only a small fraction of the underlying genetics [9].

Several theories have been proposed regarding the mechanism underlying the development and progression of idiopathic scoliosis [5]. The inherent shape of the spine may be part of the susceptibility to scoliosis [10]. The paravertebral muscles play a crucial role in spinal stability, and imbalance in these muscles may contribute to the development of idiopathic scoliosis [5]. The convex side has larger muscle volume and type I muscle fibers, while the concave side exhibits greater fatty infiltration and type II fibers, according to both histological and radiological studies [11].

These observations led us to hypothesize that there might be differences in gene expression related to muscle and adipose tissue development between the concave and convex sides of the scoliotic spine, as well as between scoliosis cases and controls without scoliosis. We therefore aimed to study gene expression differences, with special emphasis on pathways related to muscle, adipose, cartilage and bone development.

## 2 Results

### 2.1 Patient Characteristics

**FIGURE 1:**
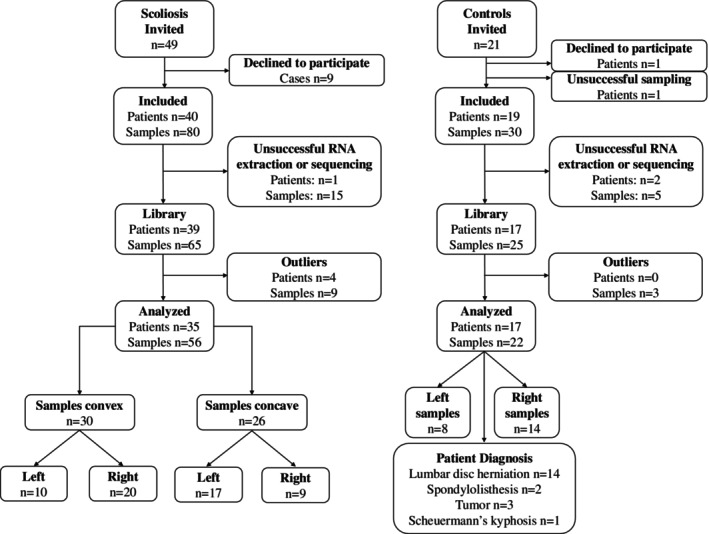
Flowchart of patient inclusion and exclusion.

In the final analysis, 35 scoliosis cases (24 females) and 17 controls (5 females) with a median age of 16 years (range 12–21 years) were included (Figure 1). Mean age (SD) at surgery was 15.9 (2.4) years for the scases and 17.1 (2.2) years for the controls. The mean major curve size Cobb angle in the scoliosis cases was 59 (10) degrees, and the major curves were thoracic (*n* = 25), thoracolumbar (*n* = 5), and lumbar (*n* = 5). Sampling level ranged from the T5/T6 disc level to the L4 vertebra for the scoliosis cases and from the T11/T12 disc level to the S1 vertebra in the controls (Table S1). Among the scoliosis cases, three patients had juvenile‐onset scoliosis (defined as onset between 4 and 9 years of age), whereas 29 patients had adolescent‐onset scoliosis (onset > 10 years). Data on age of onset were unavailable for three patients.

### 2.2 Radiological Analysis

**FIGURE 2:**
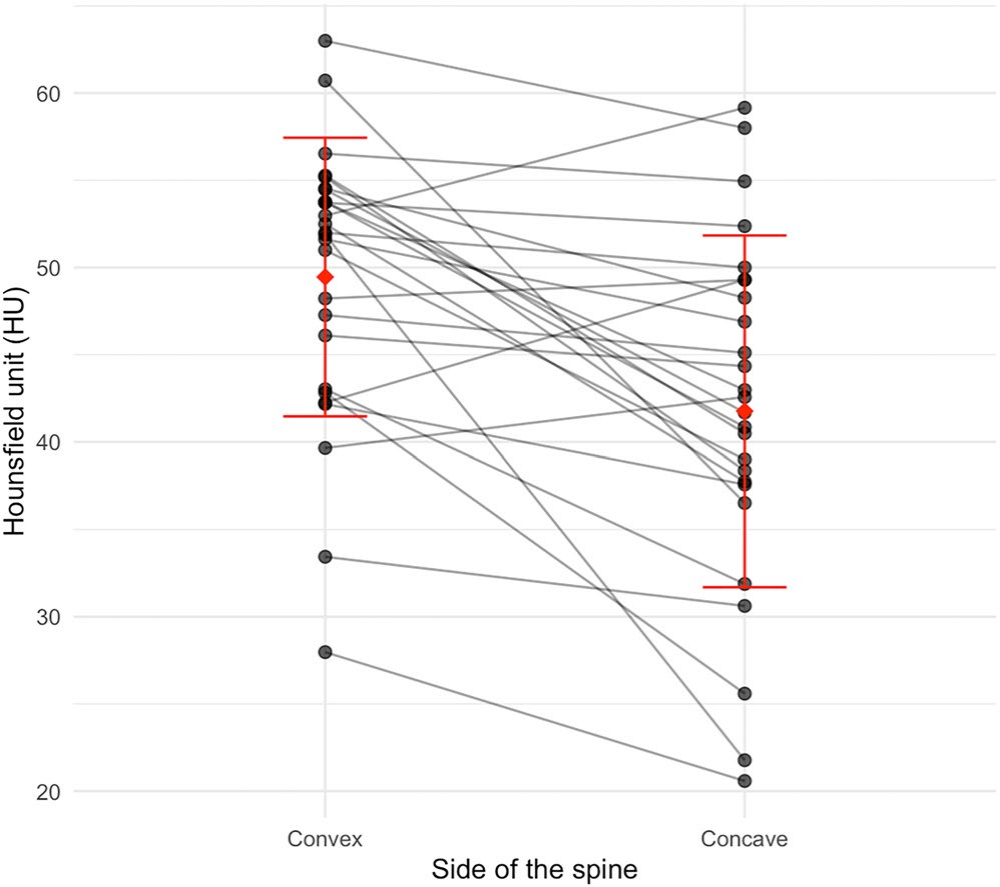
Paired dot plot showing Hounsfield unit (HU) of the paravertebral muscle at the level of sampling.

Mean Hounsfield unit (HU) of the paravertebral muscle at the level of sampling on the convex side and concave side is presented in Figure 2. The convex side HU was significantly higher, 49 (8) compared with the concave side, 41 (10), *p* = 0.0037, suggesting more muscle tissues on the convex side and more adipose tissue on the concave side.

### 2.3 Overview of Gene Expression Profiling

After removing outliers during quality control, 78 of the initial 96 samples were kept for further analysis (Figure S1). Of these, 56 muscle samples (26 concave and 30 convex) were from the scoliosis cases group, and 22 samples were from the control group.

After removing genes with low expression levels, a total of 14 212 genes were retained for downstream analysis. Among these, 7807 fluctuating genes were identified based on their higher variability compared to technical variation of added spike‐in synthetic RNAs, and their adjusted *p* values (*p*_adj_) were used for further analyses. Principal component analysis (PCA) showed a bias between the two STRT libraries, which was still present after library bias correction (Figure S2). Therefore, library information was included as a covariate factor in the differential gene expression (DGE) model.

### 2.4 Scoliosis Muscle Shows Shift in Immune and Progenitor Cell Signature

**FIGURE 3:**
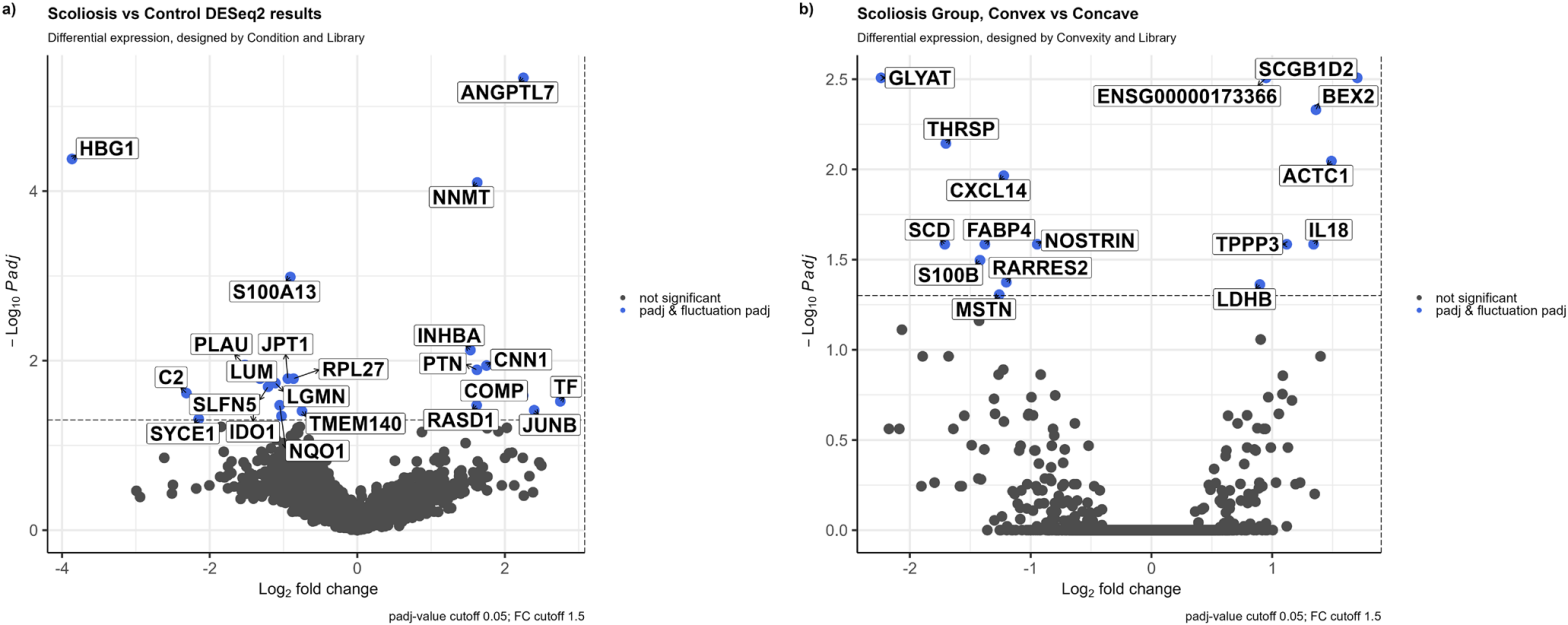
Gene expression profiles in scoliosis vs. control and convex vs. concave conditions. (A) Comparison between scoliosis and control samples, with genes highlighted in blue if they have both *p*_adj_ < 0.05 and fluctuation *p*_adj_ < 0.05, including significantly changed and fluctuated genes. (B) Comparison between convex and concave conditions, with significantly changed genes (*p*_adj_ < 0.05 and fluctuation *p*_adj_ < 0.05) also highlighted in blue. The horizontal dashed line at −log10(0.05) = 1.301 shows the significance threshold for the *p*_adj_. Gray dots are genes that are not statistically significant with *p*_adj_ > 0.05 and |log2FC| < 1.5.

In the comparison between scoliosis cases and control groups, 22 significantly differentially expressed genes (DEGs) were identified *p*_adj_ < 0.05 and fluctuation *p*_adj_ < 0.05, (Figure 3a), including genes associated with extracellular matrix remodeling such as *ANGPTL7* (up), *COMP* (up), *RASD1* (up), *LUM* (down), *PTN* (up), *PLAU* (down), *and INHBA* (up); immune regulation and stress responses *including JUNB* (up), *IDO1* (down), *C2* (down), *S100A13* (down), *NQO1* (down), *LGMN* (down) and *NNMT* (up); developmental regulation including *INHBA* (up), *SYCE1* (down) and *SLFN5* (down); and cell proliferation including *CNN1* (up), *PTN* (up), *TF* (up), *JPT1* (down) and *SLFN5* (down). In addition, fetal hematopoiesis marker *HBG1* (down) was differentially expressed (Table S4).

To test whether the transcriptomic changes in scoliosis are linked to specific cell types, we performed cell type enrichment analysis using DEGs. The gene set enrichment analysis (GSEA) showed a general decrease in immune and progenitor cell signatures in non‐fetal scoliosis muscle, while fetal Schwann cell signatures were increased (Table 1). These results indicate that scoliosis muscle is characterized by changes in immune processes and altered cell‐population signatures.

**TABLE 1:** Enriched cell types in scoliosis versus control comparison.

Direction	Cell types	*p*_adj_	NES
Down in scoliosis			
	Skeletal muscle T cells	2.53e‐10	−1.68
	Skeletal muscle B cells	2.20e‐07	−1.58
	Skeletal muscle satellite cells	2.87e‐05	−1.38
	Skeletal muscle FBN1 FAP cells	4.26e‐04	−1.36
	Skeletal muscle myeloid cells	6.00e‐04	−1.31
	Skeletal muscle PCV endothelial cells	1.77e‐03	−1.35
	Skeletal muscle NK cells	0.012	−1.30
	Fetal muscle skeletal muscle cells	0.013	−1.33
	Skeletal muscle FAP cells	0.036	−1.28
Up in scoliosis			
	Fetal muscle Schwann cells	3.77e‐06	2.22

*Note:* This table shows cell type ontology analysis results of all genes, as well as upregulated (log2FC > 0) in scoliosis and downregulated (log2FC < 0) based on log2FC and *p* value.

Abbreviations: FAP, fibro‐adipogenic progenitors; NES, normalized enrichment score; padj, adjusted *p* value using Benjamini–Hochberg correction for multiple hypothesis testing; PCV, post‐capillary venule.

### 2.5 Convex‐Concave Asymmetry Reflects Developmental Imbalance

In the convex versus concave comparison, 16 DEGs were identified (*p*_adj_ < 0.05 and fluctuation *p*_adj_ < 0.05) (Figure 3b), including genes that regulate muscle cell structure and regeneration (*ACTC1* (up), *MSTN* (down), *S100B* (down), *TPPP3* (up)), adipose remodeling (*FABP4* (down), *THRSP* (down), *RARRES2* (down), *SCD* (down)), inflammatory mediators (*CXCL14* (down), *IL18* (up), *NOSTRIN* (down)), metabolic genes (*GLYAT* (*down*), *LDHB* (*up*)), and cell cycle/apoptosis regulators (*BEX2* (up)) (Table S4). On the convex side, gene signatures related to adult skeletal muscle cell types (fibro‐adipogenic progenitors (FAP), Post‐Capillary Venule (PCV) endothelial cells, endothelial cells, FBN1 FAP cells, and satellite cells from the Rubenstein dataset) were markedly downregulated, whereas fetal skeletal muscle cell types (vascular endothelial cells, skeletal muscle cells, and myeloid cells from the DESCARTES dataset) were significantly enriched (Table 2). Among fetal muscle related signatures, vascular endothelial cells and myeloid cells were downregulated, while skeletal muscle cells were upregulated. This suggests that scoliosis asymmetry may involve a selective activation of muscle‐related developmental programs on the convex side in contrast to the concave side, which may contribute to structural imbalance in scoliosis.

**TABLE 2:** Enriched cell types in convex versus concave comparison.

Direction	Cell types	*p*_adj_	NES
Down in convex			
	Skeletal muscle FAP cells	9.45e‐08	−2.02
	Skeletal muscle PCV endothelial cells	4.94e‐05	−1.76
	Fetal muscle vascular endothelial cells	5.38e‐05	−2.06
	Skeletal muscle endothelial cells	3.04e‐04	−1.75
	Skeletal muscle FBN1 FAP cells	3.12e‐03	−1.54
	Skeletal muscle satellite cells	0.007	−1.45
	Fetal muscle myeloid cells	0.028	−1.46
Up in convex			
	Fetal muscle skeletal muscle cells	2.31e‐03	1.62

*Note:* This table shows cell type ontology analysis results of all genes, as well as upregulated (log2FC > 0) in convex and downregulated (log2FC < 0) based on log2FC and *p* value.

Abbreviations: FAP, fibro‐adipogenic progenitors; NES, normalized enrichment score; *p*_adj_, adjusted *p* value using Benjamini–Hochberg correction for multiple hypothesis testing; PCV, post‐capillary venule.

### 2.6 Differentially Expressed Genes Show Developmental Stage Specific and Cell Population Specific Expression Patterns

Cell‐type enrichment analysis suggested that muscle‐resident progenitor cells were enriched in both cell‐type enrichment analyses (Tables 1 and 2). To explore whether this enrichment is associated with developmental regulation, we next examined expression of differentially expressed genes across human developmental stages [12, 13]. A heatmap showing expression values across embryonic, fetal, juvenile, and adult muscle tissues showed that the majority of DEGs show dynamic expression patterns during early development (Figure S3). Notably, *RPL27, LDHB, PTN, HN1* (now *JPT1*), *LUM*, and *SCD* showed high expression in embryonic and fetal stages, whereas *CXCL14, JUNB, NNMT*, and *RASD1* had higher expression in later stages.

**FIGURE 4:**
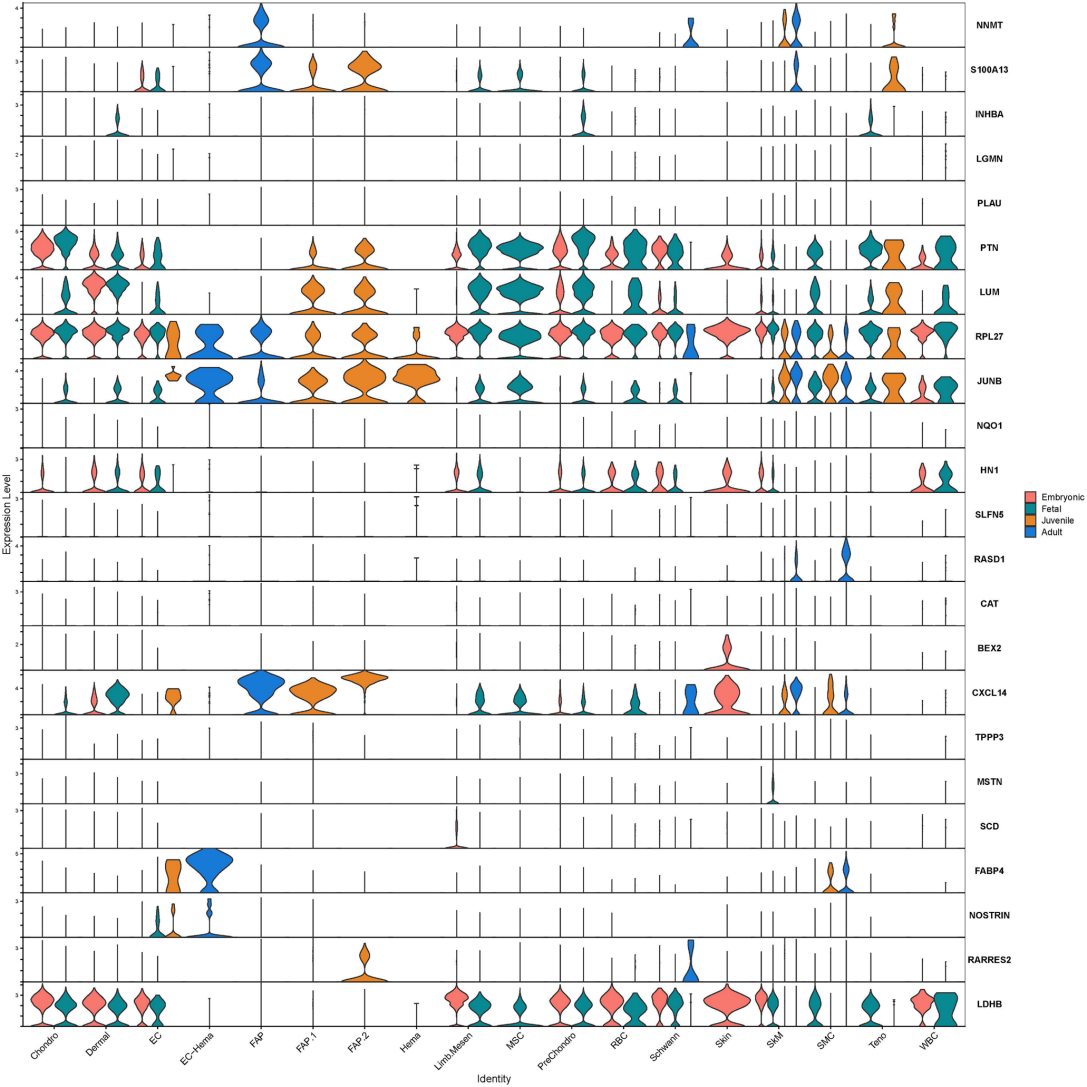
Expression of DEGs in UCSC‐Cell browser skeletal muscle dataset. Stacked violin plot showing log1p normalized expression of our DEGs across different cell types and developmental stages. Cell types are Chondro (Chondrocytes), PreChondro (Pre‐Chondrocytes), Limb.Mesen (Limb Mesenchymal Cells), MSC (Mesenchymal Stem Cells), FAP (Fibro‐Adipogenic Progenitors), Hema (Hematopoietic Cells), RBC (Red Blood Cells), WBC (White Blood Cells), Skin, Dermal, SkM (Skeletal Muscle Cells), SMC (Smooth Muscle Cells), Schwann, EC (Endothelial), and Teno (Tenocytes). Developmental stages are colored as embryonic (pink), fetal (green), juvenile (orange), and adult (blue).

To further explore the cellular specificity of these patterns, we generated violin plots separated by both developmental stage and cell type (Figure 4). Among the DEGs visualized, *PTN, LUM, RPL27, JUNB, CXCL14, LDHB*, and *S100A13* showed notable expression in mesenchymal stem cells (MSC) during fetal development. Additionally, *RPL27, JUNB, CXCL14, S100A13*, and *NNMT* showed strong expression in FAP. Genes such as *FABP4, NNMT, INHBA, RASD1, BEX2, SCD, NOSTRIN*, and *RARRES2* showed more cell population‐specific expression patterns across endothelial cells, hematopoietic cells, and Schwann cell populations. Genes such as *LGMN, PLAU, NQO1, CAT*, and *MSTN* showed very low expression (Figure 4).

These observations suggest that scoliosis‐associated transcriptional changes involve both side‐specific and developmental regulation. Such regulation is potentially driven by enrichment of several DEGs in fetal mesenchymal and progenitor cell populations such as FAPs, as well as differentiating or satellite muscle cells.

### 2.7 Sensitivity Analyses

Sensitivity analyses including sex as a covariate indicated that sex‐associated differences were largely restricted to Y chromosome genes (Figure S5), suggesting that the primary findings were robust to the inclusion of sex.

## 3 Discussion

Our study showed clear differences in gene regulation between the convex and concave sides. While the convex side was enriched for fetal skeletal muscle signatures, the concave side was enriched for adipogenic signatures (Table 2). Although these asymmetrical composition patterns between the two paravertebral muscle sides have been reported previously [11], our data extend these observations by providing additional understanding of the biological processes underlying this asymmetry.

The gene expression changes behind this asymmetry mainly involved fat‐related genes linked to fat accumulation on the concave side and muscle‐related genes linked to muscle activity on the convex side. *FABP4*, a key adipocyte marker, was upregulated on the concave side in our dataset, consistent with findings from Shu et al. [14], which showed increased *FABP4* in the paravertebral muscles of idiopathic scoliosis cases, along with impaired muscle regeneration. Similarly, *THRSP*, a regulator of *de novo* lipogenesis, was also upregulated on the concave side in our data. This finding aligns with results from Wang et al. [15], which showed increased *THRSP* expression during adipogenic differentiation of mesenchymal stem cells in adolescent idiopathic scoliosis cases. *RARRES2*, another adipokine, was upregulated on the concave side in our data. Although its direct role in scoliosis is not clear, it is known to promote adipocyte differentiation via CMKLR1 signaling [16] and it impairs insulin signaling in skeletal muscle, potentially affecting muscle function [17]. Moreover, increased circulating chemerin levels have also been observed in lipodystrophy, a condition characterized by adipose tissue remodeling [18]. *ACTC1*, a structural muscle gene, was upregulated on the convex side, and *MSTN*, a negative regulator of muscle growth, was downregulated on the convex side, suggesting different muscle activation patterns between the two sides. These transcriptomic findings are consistent with previous radiological observations from Jiang et al. who found that paravertebral muscles have greater volume on the convex side and increased fatty infiltration on the concave side in adolescent idiopathic scoliosis cases [11]. The normal paravertebral muscle Hounsfield Unit (HU) on the CT ranges from 40 to 50. In muscles with fatty infiltration, the HU is decreased since adipose tissue has a much lower HU than muscle tissues. In our radiological analysis, the HU measurement was significantly lower on the concave side compared to the convex side of the scoliosis spine, supporting the theory of more fatty infiltration on the concave side.

We also observed gene expression changes linked to extracellular matrix composition and tissue remodeling. For example, *COMP*, an extracellular cartilage oligomeric matrix protein, was upregulated in paravertebral muscle of scoliosis cases, whereas previous studies have found lower gene expression or serum levels in scoliosis [19, 20, 21]. Also, *LUM*, an extracellular matrix proteoglycan and key regulator of collagen fibrillogenesis, was downregulated in scoliosis cases compared to controls. This is consistent with previous findings showing that disrupted *LUM* function is linked to impaired collagen organization and extracellular matrix disorganization in scoliotic discs [22]. *ANGPTL7*, a regulator of extracellular matrix organization, was significantly upregulated in the scoliosis cases compared to controls in our dataset. Although its role in scoliosis has not been directly studied, findings from other skeletal conditions suggest potential relevance. Liu et al. [23] showed that ANGPTL7+ chondrocytes promote angiogenesis via FGF2‐FGFR2 signaling and contribute to cartilage degeneration in a mouse model of knee osteoarthritis. Together, these findings highlight dysregulation of extracellular matrix remodeling in scoliosis, in concordance with recent reports [24].

The findings raise the question of whether transcriptional changes are the result of the scoliosis itself or vice versa. One theory suggests the involvement of progenitor cells in embryogenesis and fetal development, resulting in an imbalance between adipocytes, myoblasts, chondrocytes and osteoblasts and eventually to the scoliosis phenotype [5, 25]. To identify specific cell populations contributing to the observed gene expression patterns, we performed cell population‐specific enrichment analysis using fetal [26] and adult muscle datasets [27].

In the scoliosis versus control comparison, we observed downregulation of several immune cell types including T cells, B cells, satellite cells, FBN1 FAP cells, myeloid cells, PCV endothelial cells, NK cells, FAPs (from the adult skeletal muscle dataset by Rubenstein et al.). In contrast, fetal Schwann cells were upregulated, and fetal skeletal muscle cells were downregulated (from the fetal dataset by Cao et al.) (Table 1).

In the convex versus concave comparison, we observed a similar trend with downregulation of FAPs, PCV endothelial cells, endothelial cells, FBN1 FAP cells, and satellite cells (from the adult skeletal muscle dataset by Rubenstein et al.). In contrast, fetal vascular endothelial cells and fetal myeloid cells were downregulated, and fetal skeletal muscle cells were upregulated (from the fetal dataset by Cao et al.) (Table 2). Supporting this, Dai et al. [28] found that a shift toward adipogenic differentiation in congenital scoliosis may be triggered by epigenetic modulation of mesenchymal stem cell differentiation via the FTO‐MMP1‐ERK pathway. Similarly, Xin et al. linked decreased ESR1 expression in concave side muscle progenitor cells to greater fatty infiltration and scoliosis severity [29], supporting the adipogenic shift observed in our study. In our dataset, fetal Schwann cell signatures were positively enriched in the scoliosis cases. This contrasts with the morphological reduction of Schwann cells in the scoliosis cases shown by Repko et al. [30], although that study did not reflect the same approach and cell developmental stage. We also observed increased satellite cell gene expression on the concave side, in line with recent finding of higher PIEZO2^+^ satellite cell numbers on the concave side in adolescent idiopathic scoliosis [31] and mechanical stretching being known to activate satellite cells [32]. In addition, *CXCL14* was upregulated on the concave side, consistent with its roles in muscle regulation. This chemokine has been shown to be enriched in fetal and juvenile muscle‐related cells. Previous research has shown that *CXCL14* negatively regulates skeletal muscle regeneration by preventing myoblasts from exiting the cell cycle, which is necessary for differentiation [33]. This effect may be more pronounced with aging and has been linked to reduced muscle repair capacity [34]. The altered expression of *CXCL14* in our data may reflect a similar mechanism, where muscle regeneration or differentiation is disrupted, potentially contributing to the observed asymmetry between the two sides of the spine. Finally, genes associated with T cells, B cells, myeloid cells and NK cells were downregulated in scoliosis samples, suggesting reduced immune activity in the paravertebral muscle tissue. This finding supports that immune dysregulation contributes to the asymmetric tissue remodeling observed in scoliosis.

To better understand whether the observed transcriptional asymmetry may originate from developmental processes and to examine the patterns of gene regulation across developmental stages, we analyzed the expression of DEGs across embryonic, fetal, juvenile and adult stages using the UCSC Cell Browser data [12, 13]. We observed that *CXCL14, JUNB, LDHB, LUM, PTN, RPL27* are highly expressed during specific fetal stages and in specific cell types. Notably, *JUNB* has been previously identified as part of a key regulon across multiple developing organs between PCW3–PCW8 in the human embryonic organogenesis atlas (Pan et al. [35], their Table S4), highlighting its early developmental regulatory role. Similarly, *ACTC1*, a structural muscle gene, was upregulated on the convex side. Although *ACTC1* itself was not specifically discussed in the recent human embryonic organogenesis atlas [35], closely related members of actin family such as *ACTA1*, *ACTB* and *ACTG1* showed specific temporal expression patterns during human muscle and cardiac development at post‐conceptional weeks (PCW) 3 to PCW8 [35] (Pan et al. their Figure 2K). In addition, findings from Pan et al. showed that *LDHB* (lactate dehydrogenase), while it has not previously been associated with scoliosis, emerges and reaches its peak expression during early organ development at PCW3‐5 [35] (Pan et al. their Figure 2L), especially in the liver. These expression pattern differences show that several DEGs are active during early developmental stages, while also potentially reflecting tissue remodeling and adaptive responses in mature skeletal muscle tissue.

To further investigate whether the observed transcriptional asymmetry may reflect developmental regulatory programs, as the STRT method captures transcription start site regions (TSS)‐associated regions including 5'UTR and 500 bp upstream, we additionally retrieved the promoter‐level expression pattern of the identified DEGs using FANTOM5 Human hg19 promoterome through the ZENBU platform [36, 37]. Our analysis showed that the identified DEGs exhibit expression patterns enriched in mesenchymal, adipogenic, stromal and developmental cellular contexts, while adaptive remodeling processes in mature skeletal muscle and adipose tissues are also likely to contribute. This finding supports the possibility that the observed transcriptional signature reflects a combination of developmental and adaptive tissue remodeling processes.

Similar developmental timing patterns have been reported in previous studies for adolescent idiopathic scoliosis. For example, Burwell et al. proposed a Cascade Concept that scoliosis may originate from disruptions in cell types of different embryonic origins, including adipocytes and osteoblasts derived from mesenchymal stem cells, and myoblasts potentially triggered by environmental or epigenetic factors [25]. Similarly, Zaydman et al. suggested that altered Pax3‐mediated neural crest cell migration may contribute to early musculoskeletal imbalances [38], while Jinnah et al. proposed that asynchronous neuro‐osseous growth mechanisms originating in early developmental asynchrony may play a central role [39]. Moreover, prospective studies show differences in body composition identifiable before the clinical onset of scoliosis, reinforcing the hypothesis that scoliosis‐related tissue divergence begins early in life [40].

A major strength of our study is the inclusion of a comparatively large cohort of both scoliosis cases and controls. The use of controls derived from patients with lumbar disc herniation and Scheuermann's kyphosis represents a potential limitation, as these conditions may not fully reflect healthy paravertebral muscle tissue. Disease‐related changes in muscle composition, inflammation, or mechanical loading could influence gene expression profiles and potentially attenuate or confound differences between the scoliosis and control groups. However, obtaining truly healthy paravertebral muscle biopsies from age‐matched individuals is not ethically or practically feasible. Another limitation is that samples were obtained from different levels in scoliosis cases and controls, and group differences in mean age and sex distribution. Additionally, scoliosis samples were collected from both primary and compensatory scoliosis curves. Furthermore, the interpretation of developmental‐stage‐related cell populations inferred from bulk RNA‐seq deconvolution is limited by the currently available reference datasets, which represent fetal and adult tissues rather than adolescent skeletal muscle specifically.

Taken together, our study extends previous observations of paravertebral muscle asymmetry by integrating transcriptomic, radiological and cell type enrichment analyses. We show that the convex side has a more muscle‐related pattern, whereas the concave side has a more adipose‐related pattern, associated with immune and extracellular matrix changes. Together, these findings suggest that the molecular differences observed in scoliosis reflect contributions from both developmental and adaptive remodeling processes.

## 4 Methods

### 4.1 Study Participants

The flowchart for inclusion and exclusion is presented in Figure 1. Patients with diagnosed idiopathic scoliosis, aged 10–25 years, undergoing surgery at the Karolinska University Hospital, Sweden between 2018 and 2021 were invited to participate in the study. Inclusion was done consecutively. Patients of similar age undergoing spine surgery at Karolinska University hospital for non‐scoliotic conditions participated as controls (Figure 1).

### 4.2 Radiology

The preoperative Cobb angle was determined using the last erect whole spine radiograph before surgery for the idiopathic scoliosis cases [41]. The convexity and the levels of sample collection for each scoliosis case are shown in Figure S4. For the controls, erect spine x‐ray was not always performed; however computed tomography (CT) and magnetic resonance images (MRI) were examined to rule out any scoliosis curves.

The preoperative whole spine CT was available for 26 out of 35 scoliosis cases and examined. Measurements of Hounsfield units (HU) on the paravertebral muscle on the level of muscle biopsy were performed as described by Shimoda et al. [42]. A third‐year resident in orthopedic surgery (TC) and an orthopedic spine surgeon with 25 years of experience in spinal deformity surgery (PG) made the measurements independently of each other.

Each observer made multiplanar reconstructions of the computed tomography for a “true axial” of the spine at the level of the muscle biopsies. All measurements were made with the imaging software SECTRA IDS7 version 25.2 (SECTRA, Linköping, Sweden). The mean of the two observers' measurements for HU for each patient on the convex side and concave side was calculated and presented using a paired dot plot. The Wilcoxon signed‐rank test was performed using RStudio version 2023.12.1+402.

### 4.3 Sampling

Muscle biopsies were obtained from both the concave and convex sides of the scoliosis cases during surgery. The muscle biopsies were obtained predominantly near the convexity of the major curve or in the convexity of the compensatory curve. The level of sampling and the convexity of the curve are shown in Figure S4 for all scoliosis cases. For the controls, the majority had spinal conditions requiring surgery in the lumbar spine. Since not all patients underwent surgery on both sides of the spine, a single‐sided sample was obtained when bilateral samples could not be collected (Figure S4). Muscle biopsies were obtained during surgery and transferred directly into RNA*later* solution (Invitrogen, Thermo Fisher Scientific) for stabilization. The samples were initially stored at room temperature for 24 h and subsequently stored long term in −80°C. RNA extraction and sequencing were performed at the same time for all samples.

### 4.4 RNA Extraction

Total RNA was isolated from the tissue samples using the RNeasy Lipid Tissue Mini Kit (Cat. No. 74804, Qiagen). RNA quality was assessed as RIN scores, using an Agilent BioAnalyser, with a threshold of > 6.5 for sample inclusion. Concentration of RNA was measured using Qubit.

### 4.5 RNA‐Sequencing With STRT Method

A modified 5′‐end RNA‐seq method, Single‐Cell Tagged Reverse Transcription (STRT), with 8‐bp unique molecular identifiers (UMIs) was applied [18, 43]. Forty nanograms of total RNA from each sample was used for library preparation. Briefly, RNA samples were placed in a 48‐well plate in which a universal primer, template‐switching oligos, and a well‐specific 6 bp barcode sequence were added to each well. The synthesized cDNAs from the samples were then pooled into one library and amplified by single‐primer PCR with the universal primer sequence. The resulting amplified library was then sequenced using an Illumina NextSeq500 instrument, High Output (75 cycles, single‐end). We obtained approximately ~6.0 million and ~9.6 million qualified reads per sample in Library 1 and Library 2, respectively (Tables S2 and S3). These values represent quality‐filtered reads prior to duplicate removal and therefore reflect the effective sequencing depth.

### 4.6 Preprocessing and Quality Control of the STRT Libraries

The STRTN pipeline (https://github.com/gyazgeldi/STRTN, commit e16a6d1) [44] was used to preprocess the raw STRT‐seq data. Reads were aligned to the human hg38 reference and annotated using GENCODE v43 basic annotation (wgEncodeGencodeBasicV43), which was downloaded from the UCSC Genome Browser at https://genome.ucsc.edu/ [45]. After that, for each multiplexed STRT library (48 samples per library), a gene count matrix was obtained along with the following quality control metrics: mapped reads, spike‐in reads, spike‐in 5′‐end rate, mapped rate, mapped/spike‐in ratio, and coding 5′‐end rate metrics. Based on these metrics, for two libraries, outlier samples, non‐template controls (NTCs) and technical duplicates were excluded from both libraries.

### 4.7 Reduction of Technical Biases and Noise in the Gene Expression Profiles

Genes expressed in > 5 samples were retained, and their count matrix and depth files were processed to correct for library‐specific biases between the two STRT libraries, using the Negative Binomial Generalized Linear Model—Library Bias Correction (NBGLM‐LBC) package in R (version 4.2.2) [46]. Depth files, extracted from BAM files using samtools [47], were processed and classified by library. A feature selection test was then used to identify genes with high variability compared to spike‐in noise; genes with adjusted *p* values < 0.05 were considered fluctuating [43]. Principal Component Analysis (PCA) was performed on both the raw count matrix and library bias corrected count matrix using the Seurat package (version 5.0.1) in R to identify library bias, followed by spike‐in normalization with log transformation [48].

### 4.8 Differential Gene Expression (DGE) Analysis

DGE was analyzed using the DESeq2 package (version 1.38.3) [49] with Wald test in R. Spike‐in normalization was performed by specifying the spike‐ins in the controlGenes parameter. The comparisons included 56 scoliosis samples versus 22 control samples, and 30 convex versus 26 concave side samples within the scoliosis group. Library information was used as a covariate factor in the model design. The results were visualized using the EnhancedVolcano package (version 1.16.0) (Blighe, 2018/2024) in R.

### 4.9 Gene Set Enrichment Analysis (GSEA)

GSEA was performed using curated cluster markers for cell types from the C8 collection, to identify enriched cell types in the sample groups [50, 51, 52]. The ranking of genes based on log2FC and *p* value was analyzed using the fgsea package (version 1.24.0) in R [53]. For this analysis, gene sets related to muscle and fetal muscle development were selected from “DESCARTES FETAL MUSCLE” (fetal tissues) [26] and “RUBENSTEIN SKELETAL MUSCLE” (adult skeletal muscle, mononuclear cell populations) [27] datasets. The Developmental Single Cell Atlas of gene RegulaTion and ExpresSion (DESCARTES) dataset profiles a single‐cell atlas of human fetal tissues (72–129 days post‐conception) across 15 developing organs, including fetal skeletal muscle. The RUBENSTEIN dataset profiles adult skeletal muscle (aged 24–84), combining RNA‐seq of isolated Type I and Type IIa muscle fibers with single‐cell RNA‐seq of mononuclear cells such as fibro‐adipogenic progenitors, satellite cells, endothelial, and immune cells from vastus lateralis biopsies.

### 4.10 Visualization

Datasets were downloaded from UCSC Cell Browser—the Skeletal Muscle [12] for embryonic development hindlimb muscle (weeks 5–6, 6–7 and 7–8), fetal development hindlimb muscle (weeks 9, 12–14 and 17–18), juvenile hindlimb muscle, and adult hindlimb muscle [13] to explore whether the expression of the significantly changed genes changes during muscle development. The count matrices and associated metadata were combined, and the NormalizeData function from the Seurat package was applied with default parameters to normalize this combined dataset. Following that, a heatmap and violin plot were created to illustrate the expression of differentially expressed genes in the relevant cell populations across developmental stages using ComplexHeatmap package (version 2.14.0) [54] and the VlnPlot function of Seurat. UMAPs from the UCSC‐Cell Browser website were also used to show the expression profiles across these developmental stages [12].

### 4.11 Sensitivity Analyses

Sensitivity analyses including sex as a covariate were performed.

## Author Contributions

**Sini Ezer:** formal analysis, writing – original draft, investigation. **Gamze Yazgeldi Gunaydin:** investigation, formal analysis, visualization, writing – original draft, writing – review and editing, methodology, validation, software. **Elisabet Einarsdottir:** conceptualization, methodology, data curation, investigation, formal analysis, supervision, writing – original draft, writing – review and editing. **Tian Cheng:** methodology, writing – original draft, writing – review and editing, formal analysis, investigation, data curation, visualization, validation, software. **Shintaro Katayama:** methodology, data curation, software, investigation, formal analysis, supervision, writing – original draft, writing – review and editing, visualization. **Paul Gerdhem:** conceptualization, methodology, software, data curation, investigation, validation, formal analysis, supervision, funding acquisition, visualization, project administration, resources, writing – original draft, writing – review and editing. **Juha Kere:** conceptualization, methodology, software, data curation, investigation, validation, formal analysis, supervision, funding acquisition, visualization, project administration, resources, writing – original draft, writing – review and editing.

## Funding

This study was supported by grants from the Swedish Research Council (Dnr 2012‐02275 and 2017‐0139), the Stockholm County Council (ALF‐agreement), Center for Innovative Medicine (CIMED) Karolinska Institutet, H.R.H. Crown Princess Lovisa's Association for Child Healthcare, Sigrid Jusélius Foundation and Jane, and Aatos Erkko Foundation.

## Ethics Statement

This study was approved by the Swedish Ethical Review Authority in Stockholm. Case (Dnr) number 2017/2374‐31, 2018/1457‐32, 2022/00700‐02, 2024/05452‐02.

## Consent

Written informed consents were obtained from all study participants prior to participation in the study.

## Conflicts of Interest

The authors declare no conflicts of interest.

## Supporting information


**Figure S1:** Outlier samples were excluded from further analysis based on quality control metrics across both libraries. (A) In the first library, outliers were samples 6, 15, 33 (NTC), 37, 46, 47, as well as sample 2 due to technical issues and sample 16 as a technical duplicate. (B) In the second library, outliers were samples 3, 5, 7, 8, 9 (NTC), 18, 44, 45, 48, and sample 43 as a technical duplicate.

**Figure S2:** Bias between the two libraries. (a) Bias before library correction. (b) Existing bias remaining after correction. Due to this, library information as a covariate factor was included in the DGE analysis model.

**Figure S3:** Gene expression dynamics of differentially expressed genes (DEGs) across developmental stages and cell types. Heatmap showing the normalized expression levels (log1p values) of DEGs during embryonic stages in the UCSC‐Cell Browser Skeletal Muscle Datasets. Columns represent the developmental stages: embryonic, fetal, juvenile, and adult. Rows correspond to DEGs identified in our analysis. The color scale indicates log1p normalized expression levels, with yellow representing higher expression and purple indicating lower expression. Columns are split by four developmental stages: embryonic, fetal, juvenile, and adult and represent cell types including Chondro (Chondrocytes), PreChondro (Pre‐Chondrocytes), Limb.Mesen (Limb Mesenchymal Cells), MSC (Mesenchymal Stem Cells), FAP, FAP.1, FAP.2 (Fibro‐Adipogenic Progenitors), Hema (Hematopoietic Cells), EC‐Hema (Endothelial‐Hematopoietic Cells), RBC (Red Blood Cells), WBC (White Blood Cells), Skin, Dermal, SkM (Skeletal Muscle Cells), SMC (Smooth Muscle Cells), Schwann, EC (Endothelial), and Teno (Tenocytes).

**Figure S4:** Radiographs of all patients and samples that have been successfully analyzed. The level marking indicates the level where the muscle biopsies were obtained. The triangle indicates what we determined as the convex side while the circle indicates the concave side. Samples that were not analyzed were not shown. For controls, a generic X‐ray image of the lumbar spine was used to illustrate the level and side of the sample.

**Figure S5:** Male vs. Female differential expression analysis in both Control and Scoliosis group including sex as a covariate factor showed sex differences mainly in Y‐linked genes, plus CAMP, IGLV3‐21 in Scoliosis. Red dots are genes that are statistically significant *p*_adj_ < 0.05 and |log2FC| > 1.5; while gray dots are genes that are not statistically significant with *p*_adj_ > 0.05 and |log2FC| < 1.5. Green dots are genes with |log2FC| > 1.5. The horizontal dashed line at −log10(0.05) = 1.301 shows the significance threshold for the *p*_adj_.

**Table S1:** Baseline data for idiopathic scoliosis cases and controls. Data is presented as number (percentage) and mean (standard deviation). n/a: not available.

**Table S2:** Quality control and sequencing metrics for all samples in Library 1 generated from the STRT RNA‐seq preprocessing pipeline. Provided as a separate file.

**Table S3:** Quality control and sequencing metrics for all samples in Library 2 generated from the STRT RNA‐seq preprocessing pipeline. Provided as a separate file.

**Table S4:** Differentially expressed genes in scoliosis muscle samples compared to controls and between convex and concave sides. The list includes down and up‐regulated genes with their descriptions, functions and associated cell‐types. Provided as a separate Excel file.

## Data Availability

The datasets generated in this study include sensitive information; therefore, they are not publicly available due to the agreement of informed consent. However, access to the data can be granted upon reasonable request to the corresponding author if approved by the ethics committee and the Uppsala University GDPR officer.
