# Supplementary material for: Fetal Pathogenesis of Scoliosis Suggested by Asymmetry of Gene Expression in Paravertebral Muscles
Source: JOR Spine. 2026 Aug 16;9(3):e70214. doi: 10.1002/jsp2.70214 (PMC13478252; doi:10.1002/jsp2.70214)

Patient 1 Patient 2 Patient 3 Patient 4

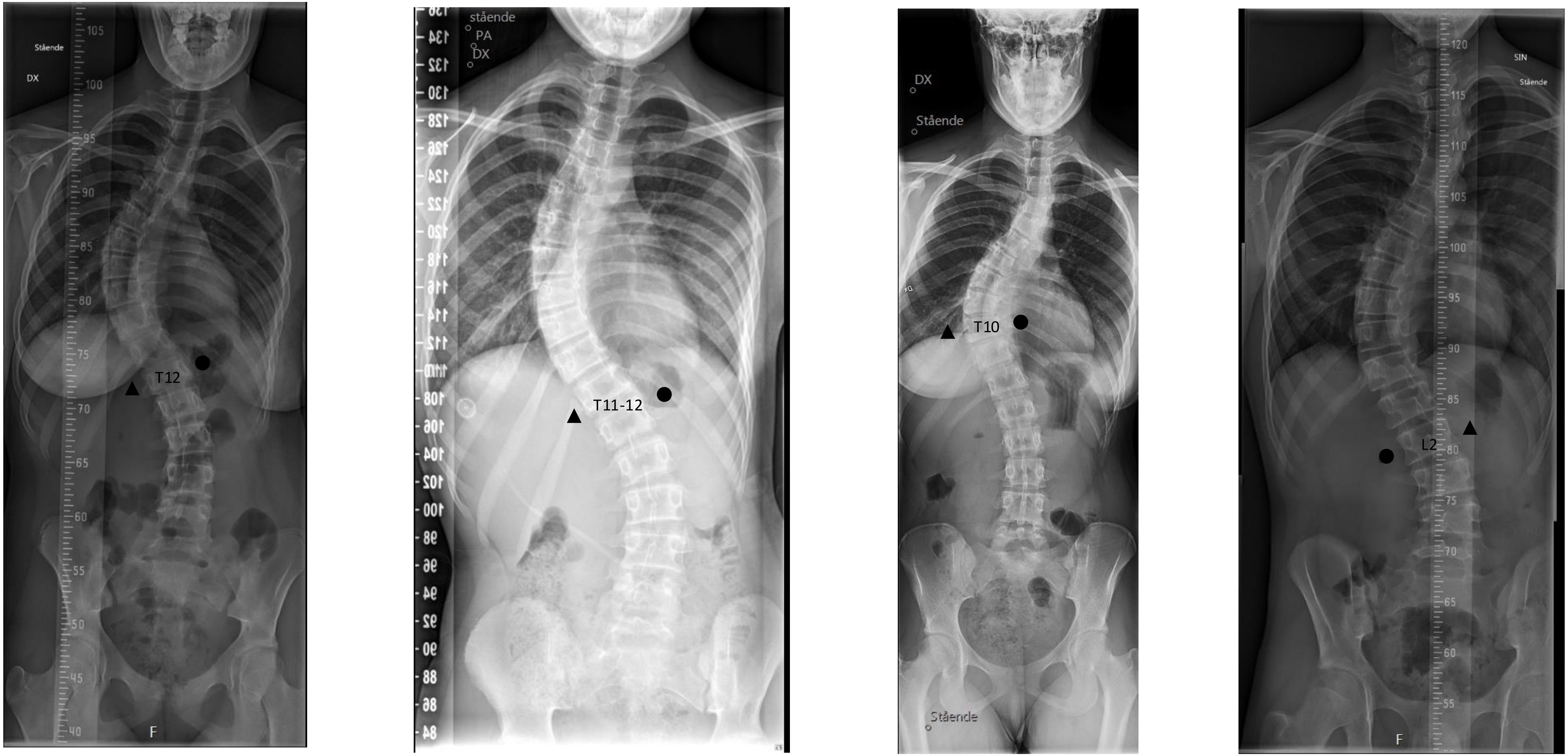


▲ Sample Level ● Triangle: Convex side
Circle: Concave side

Patient 5

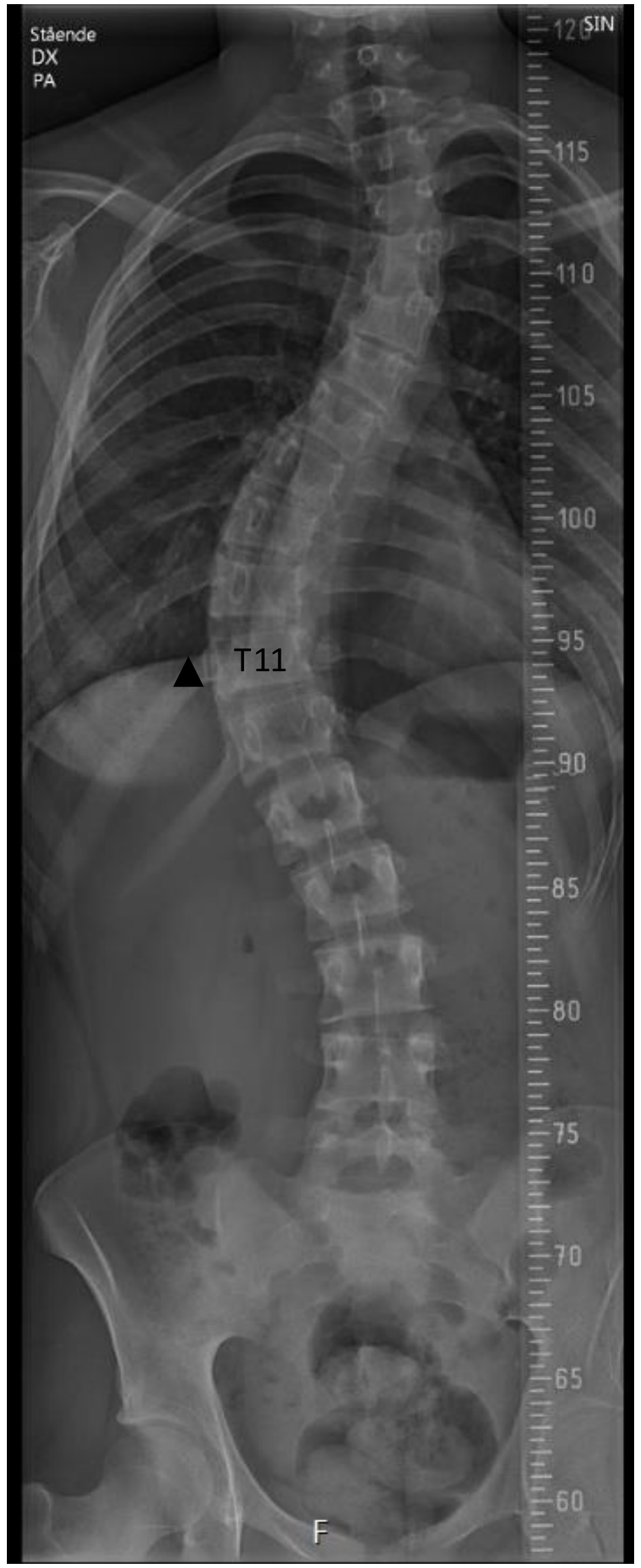


Patient 6

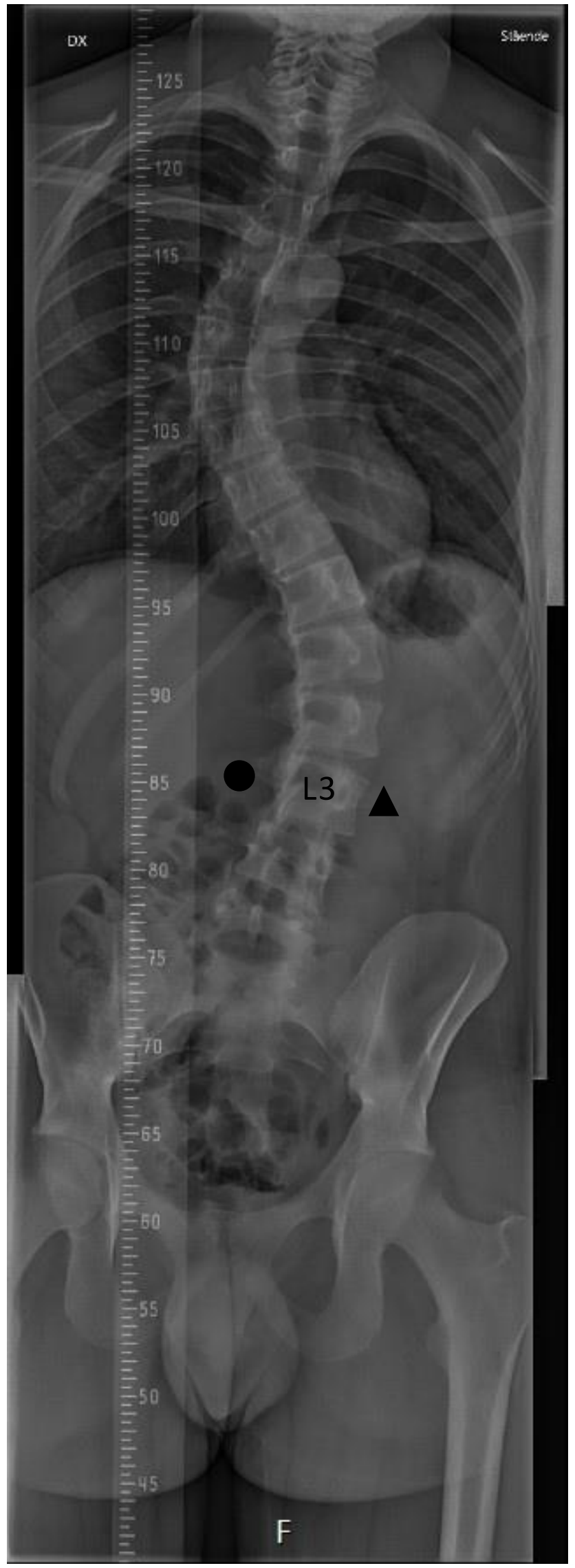


Patient 7

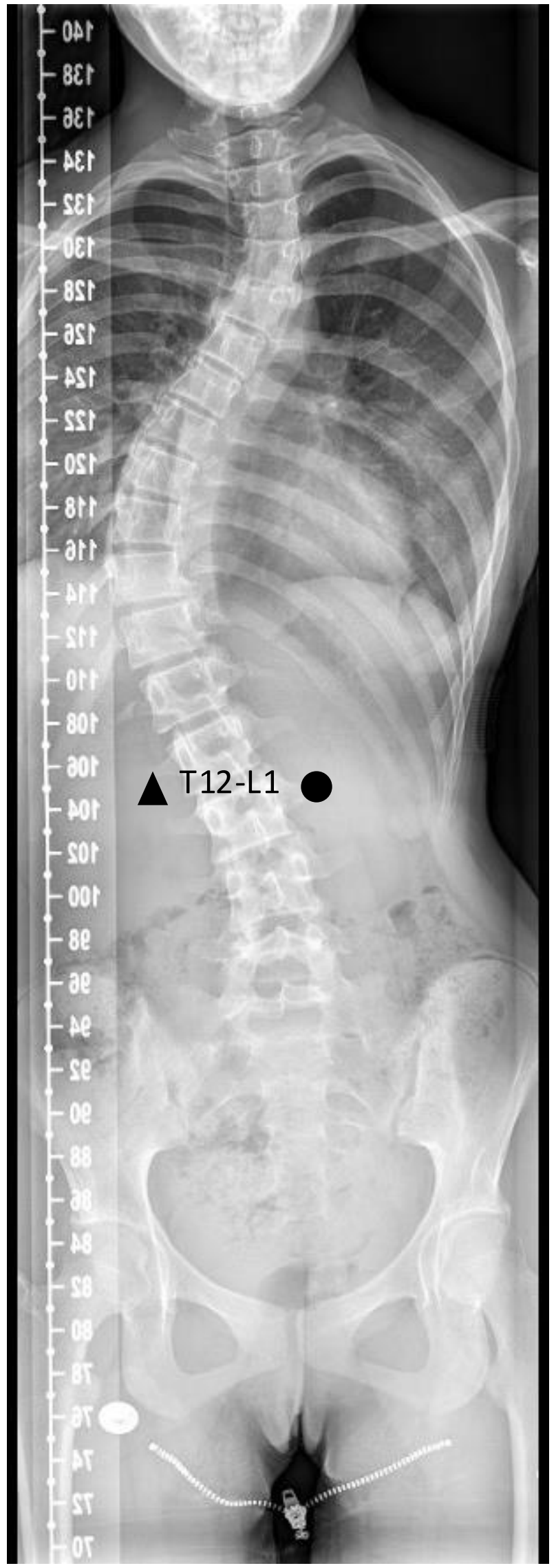


Patient 8

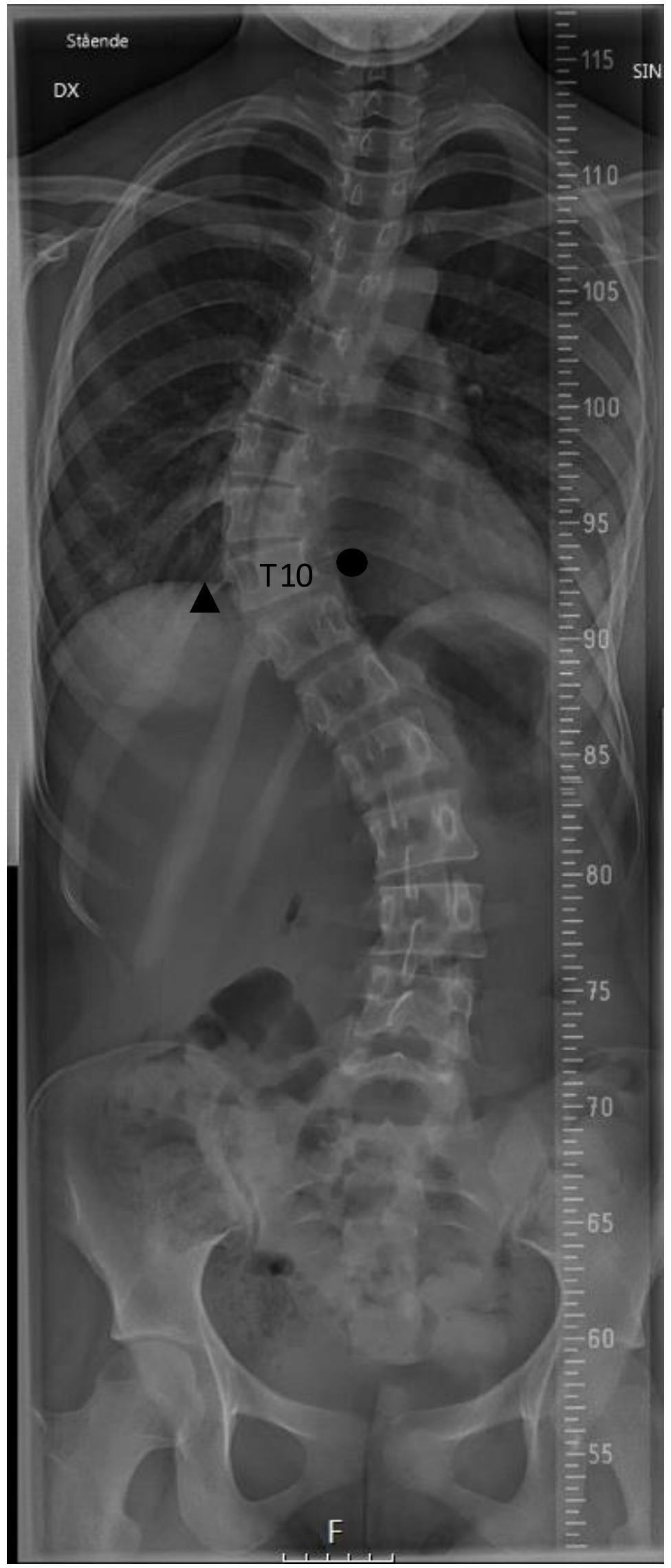

Patient 9

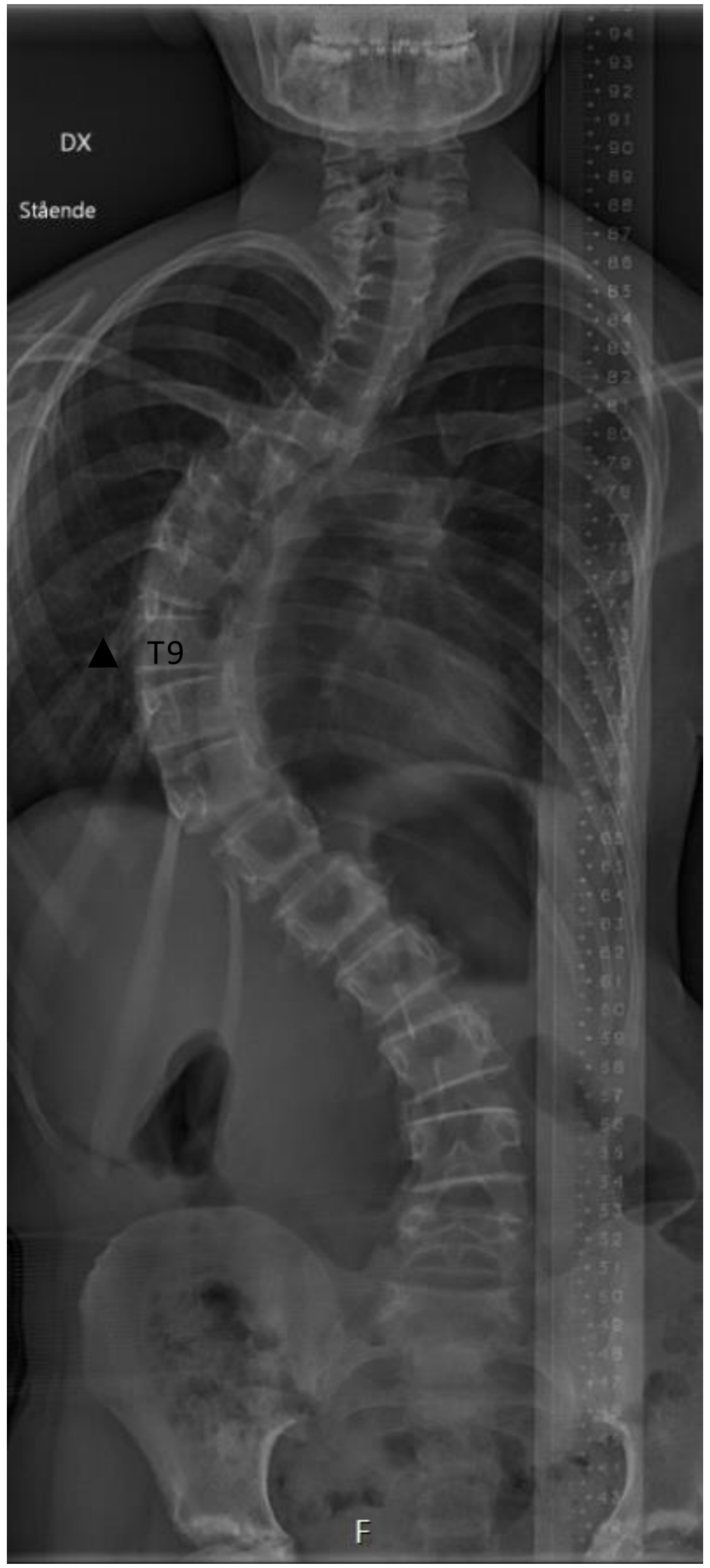


Patient 10

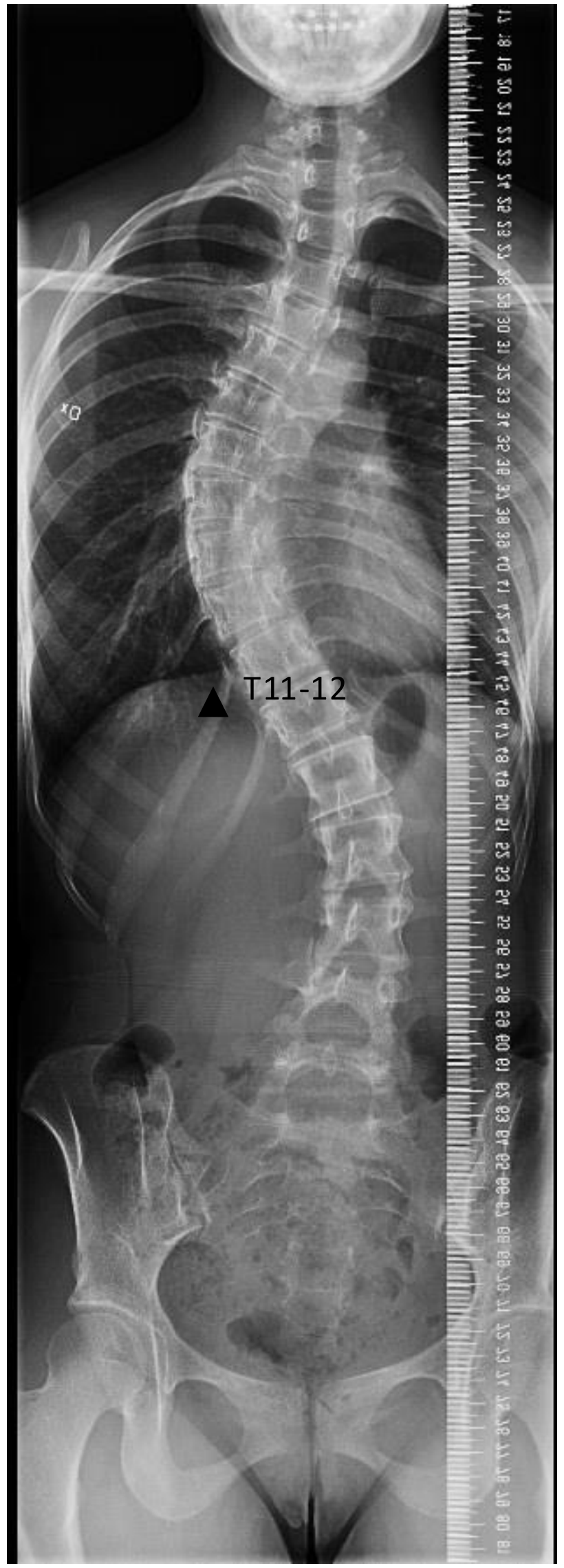


Patient 11

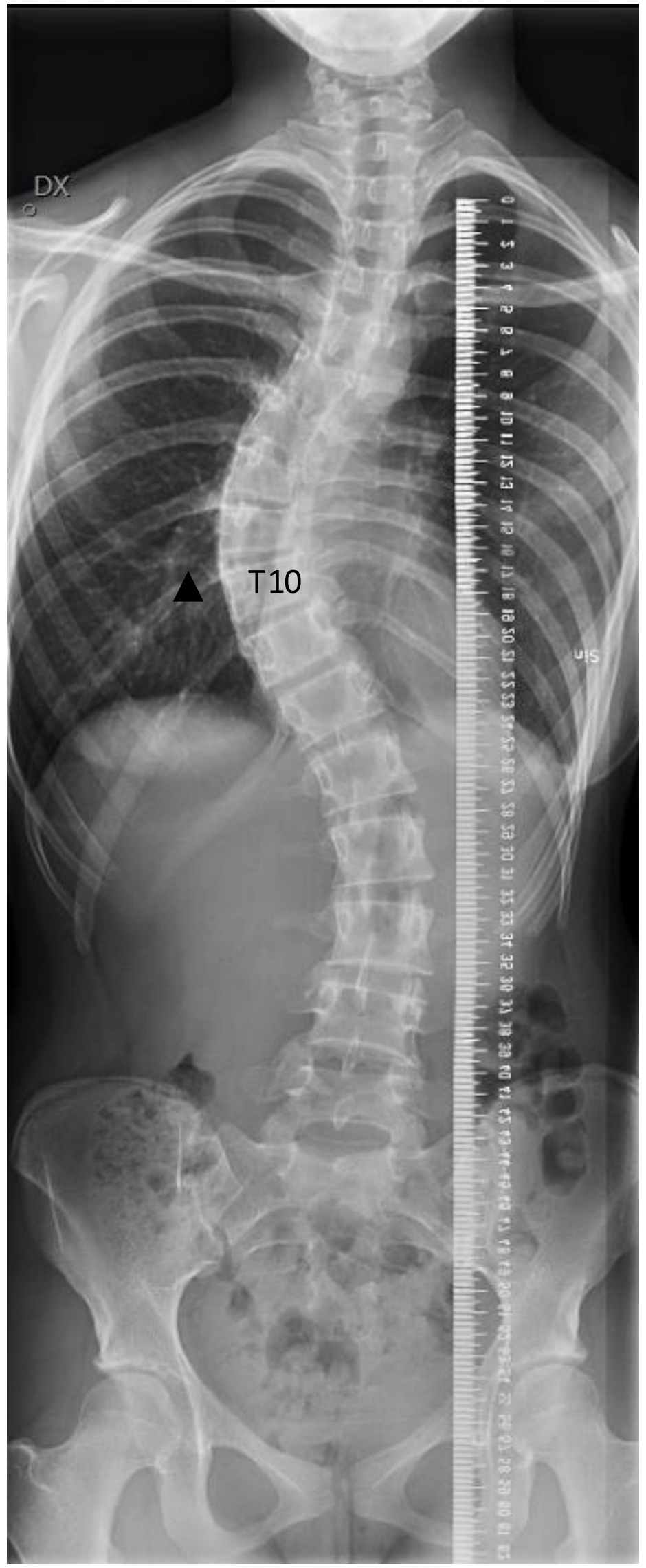


Patient 12

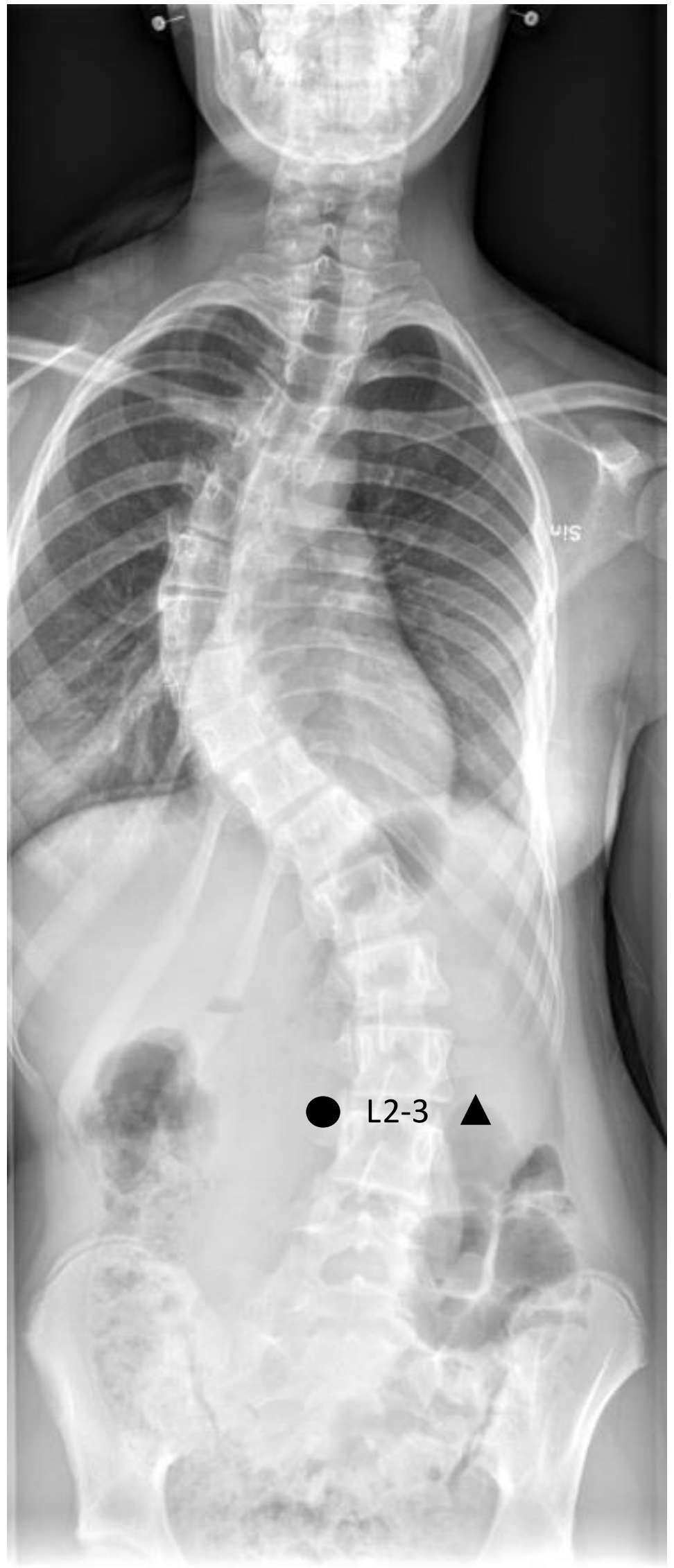

Patient 13

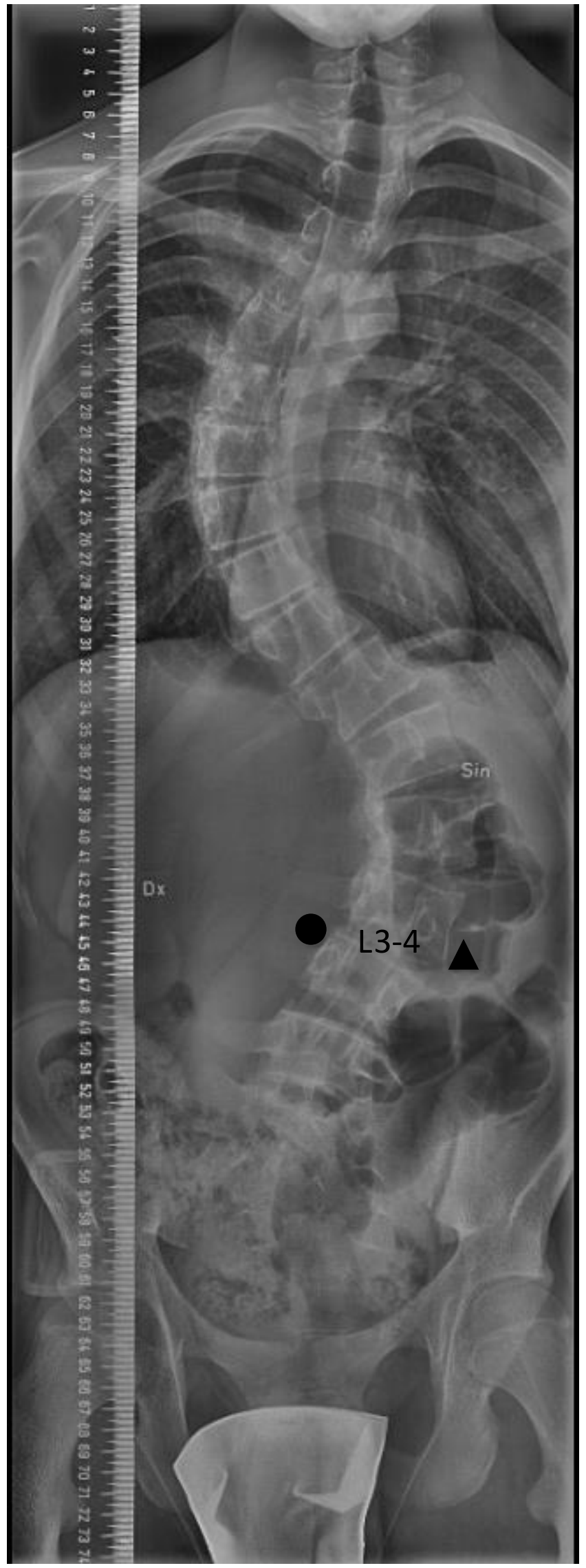


Patient 14

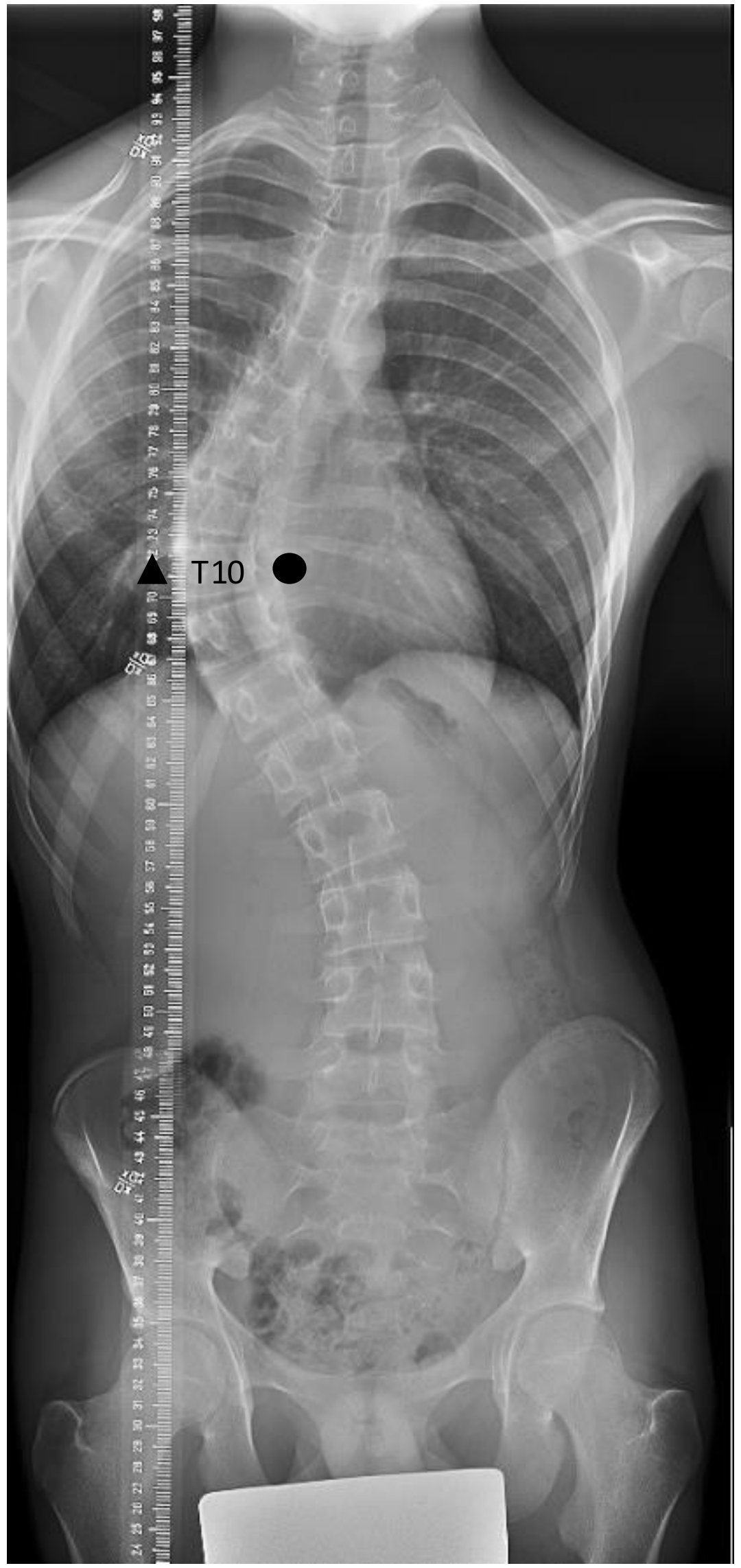


Patient 15

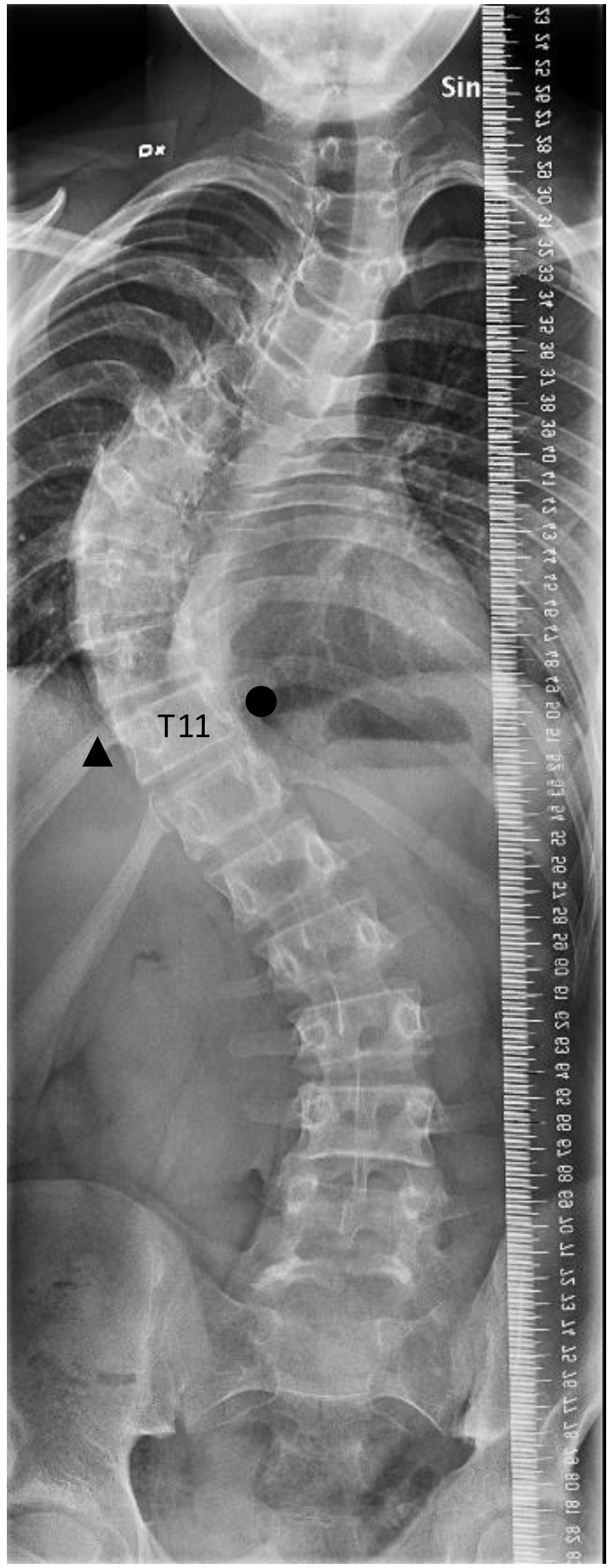


Patient 16

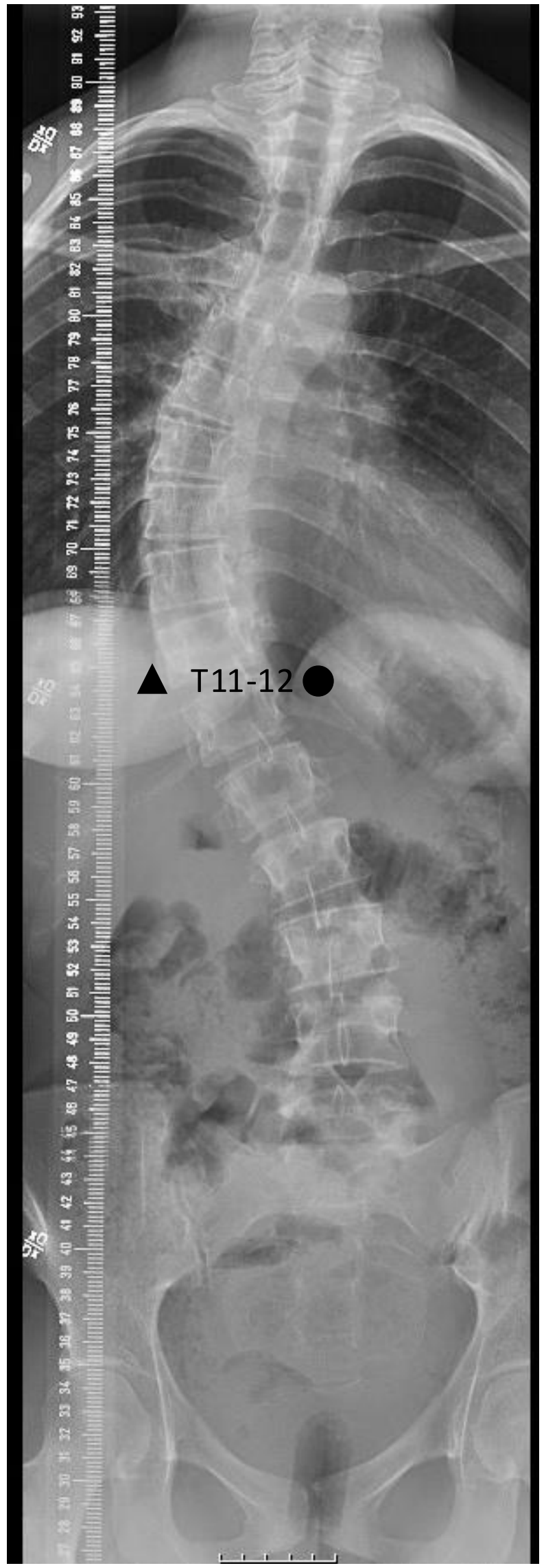

Patient 17

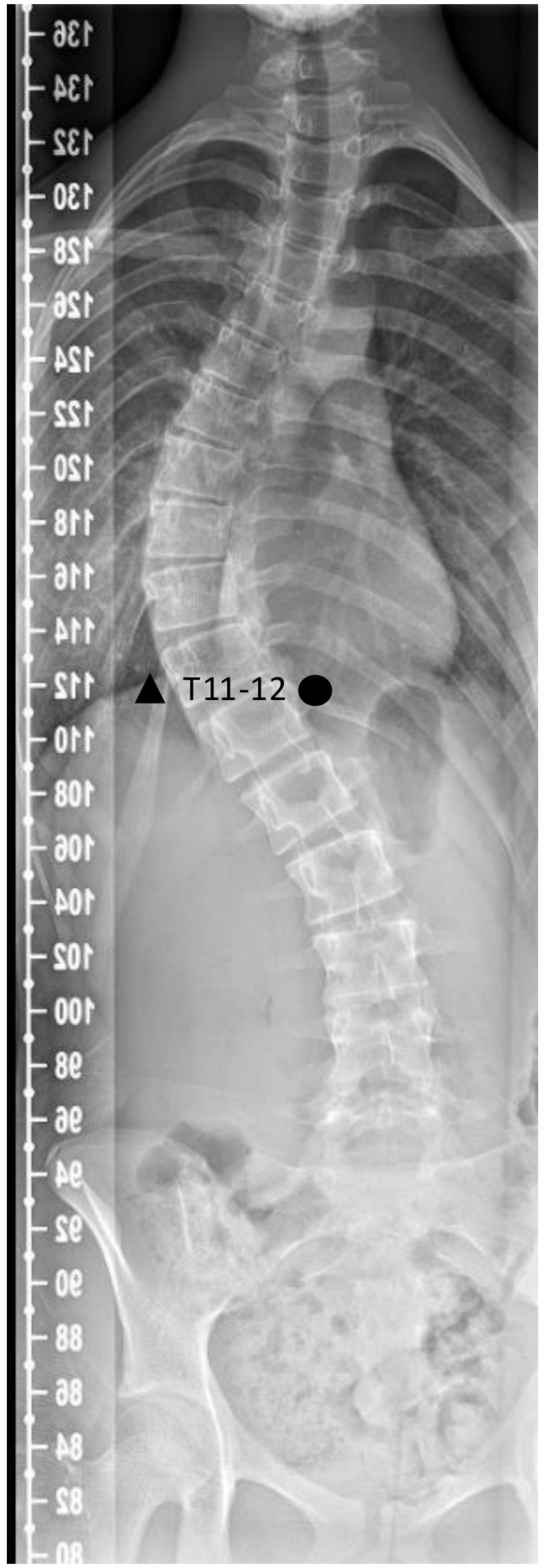


Patient 18

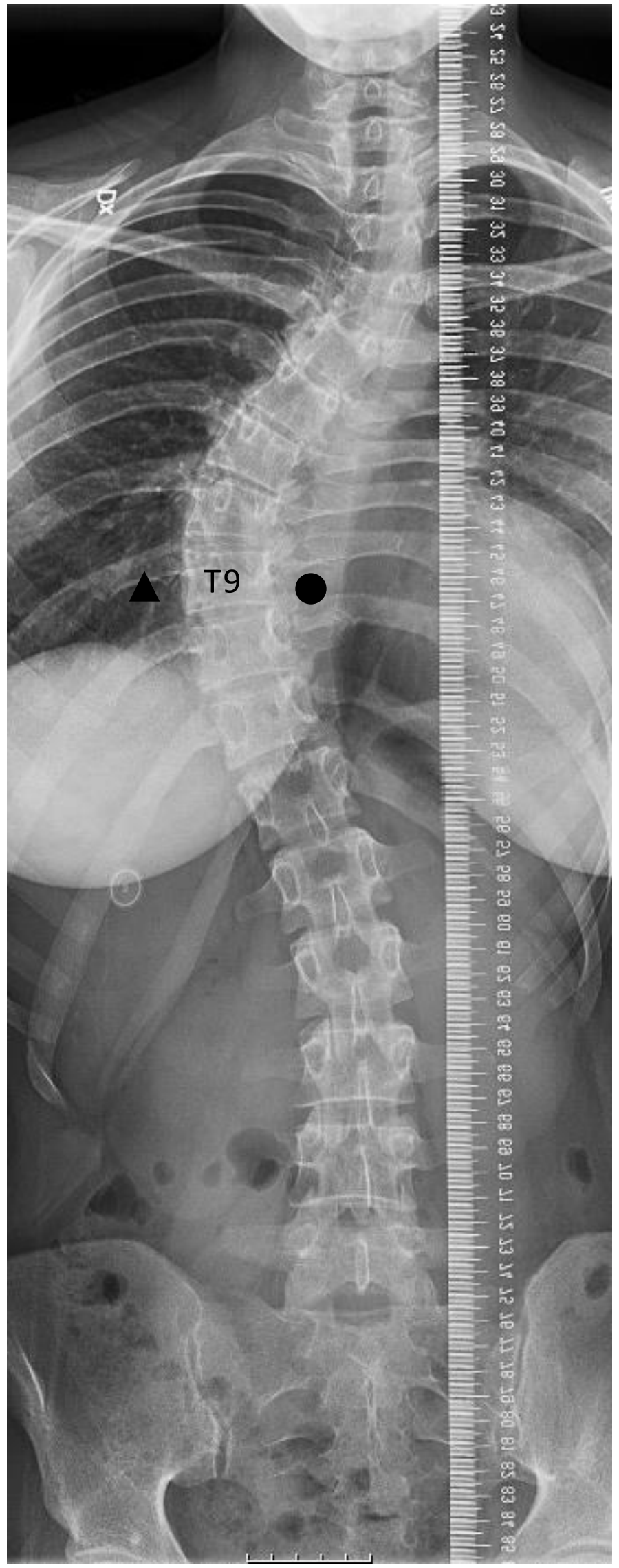


Patient 19

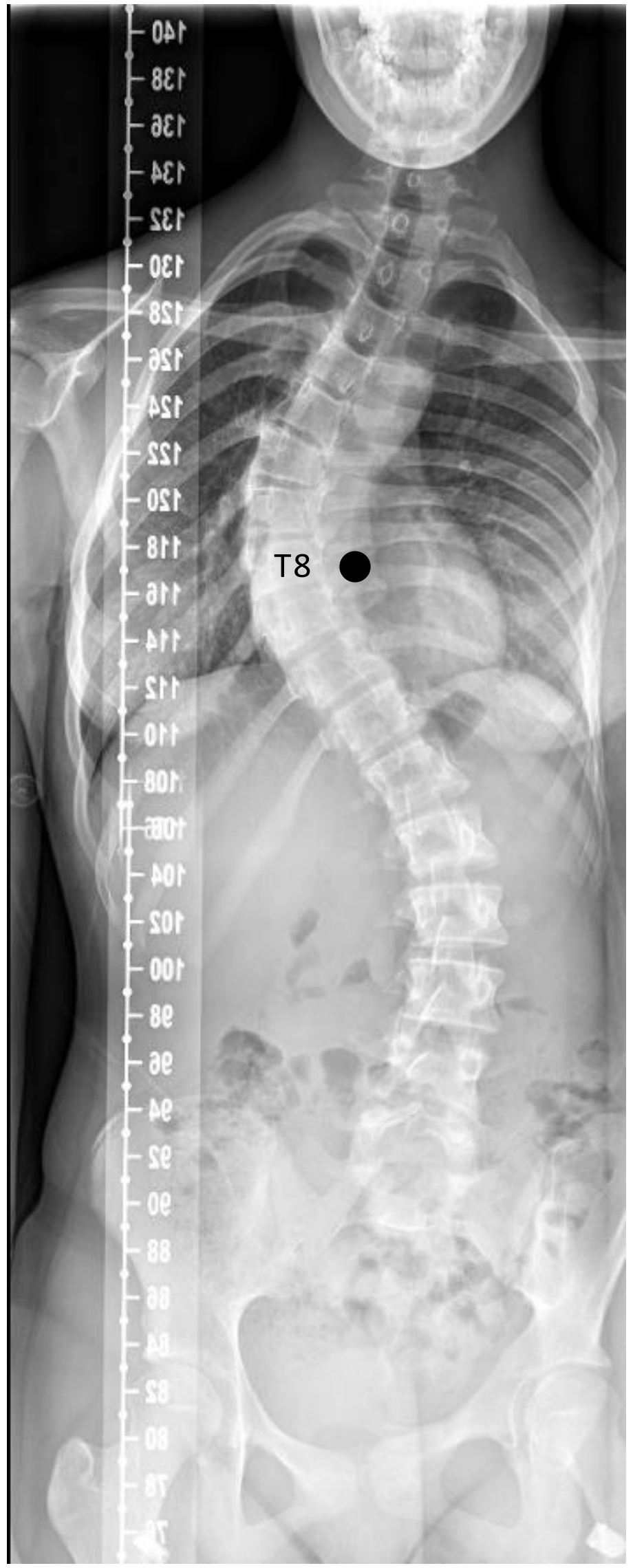


Patient 20

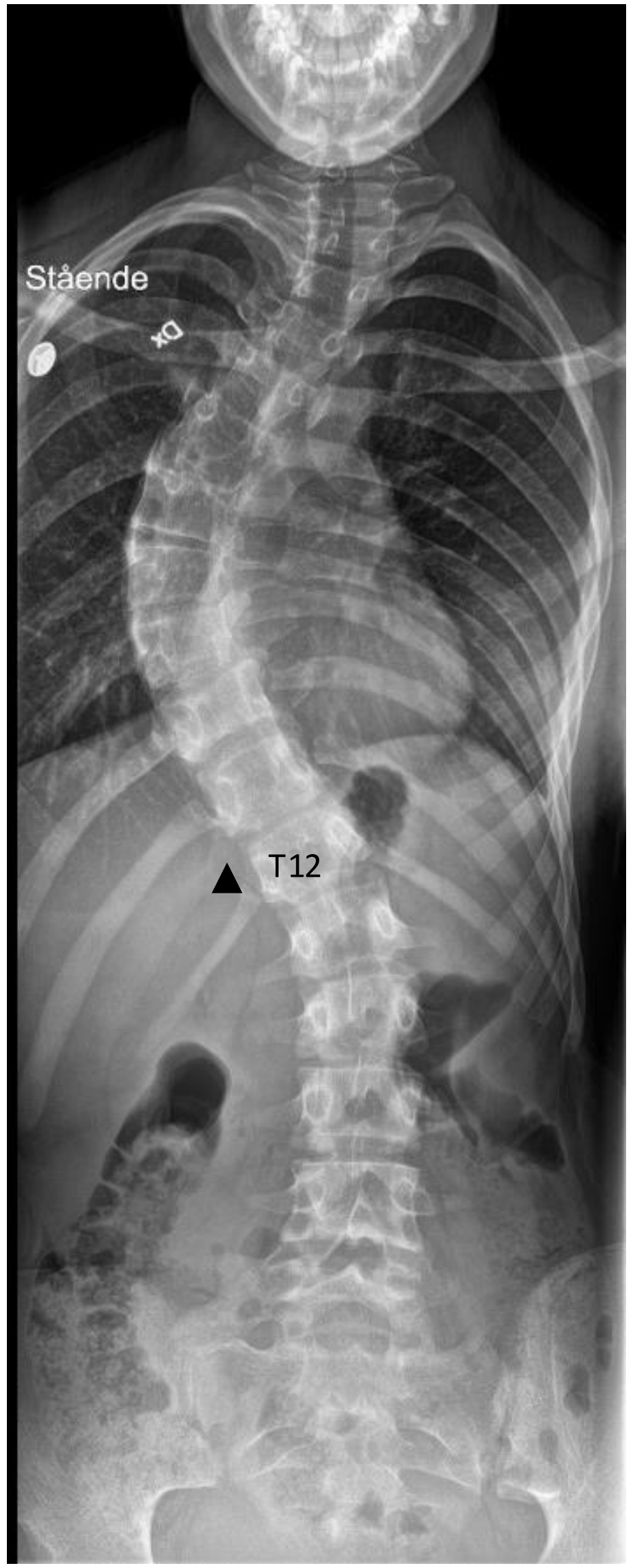

Patient 21

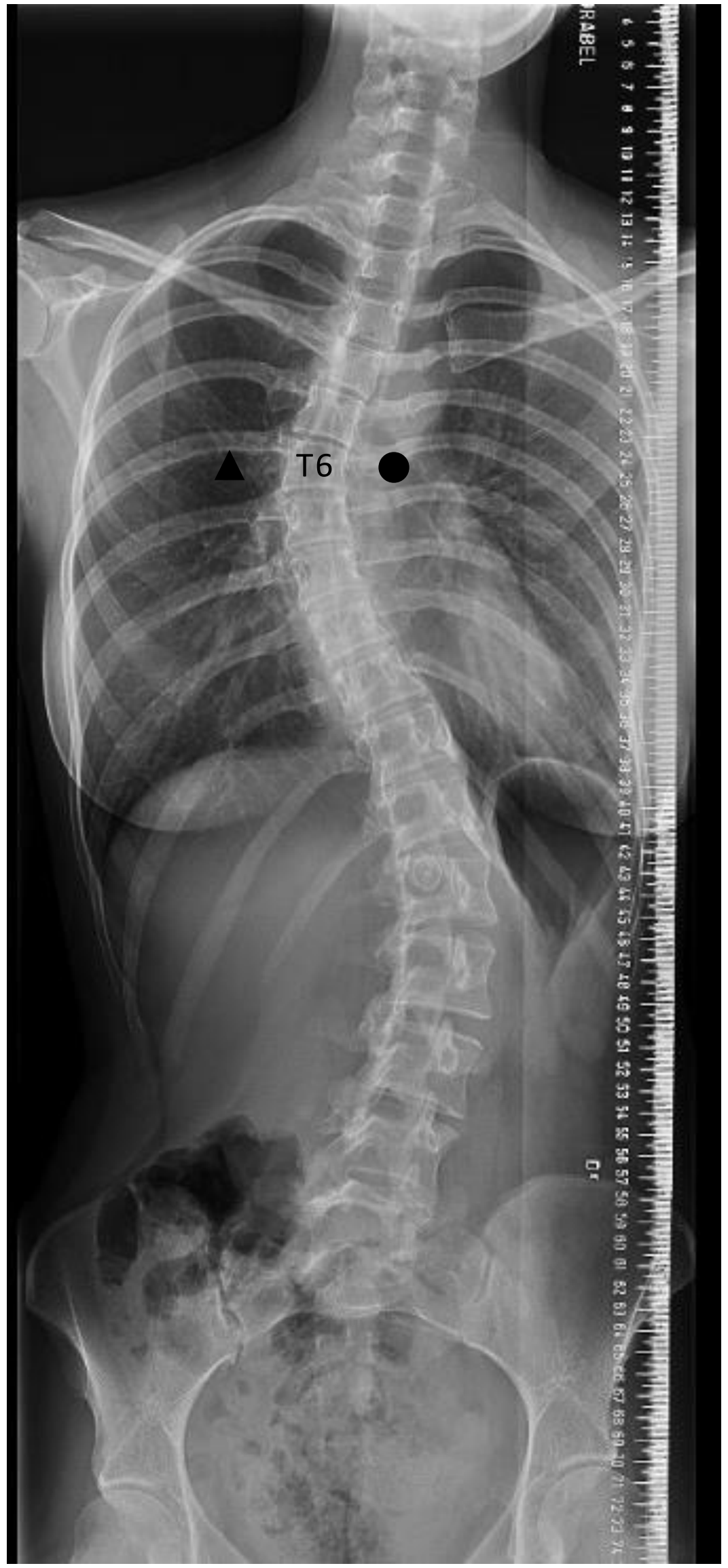


Patient 22

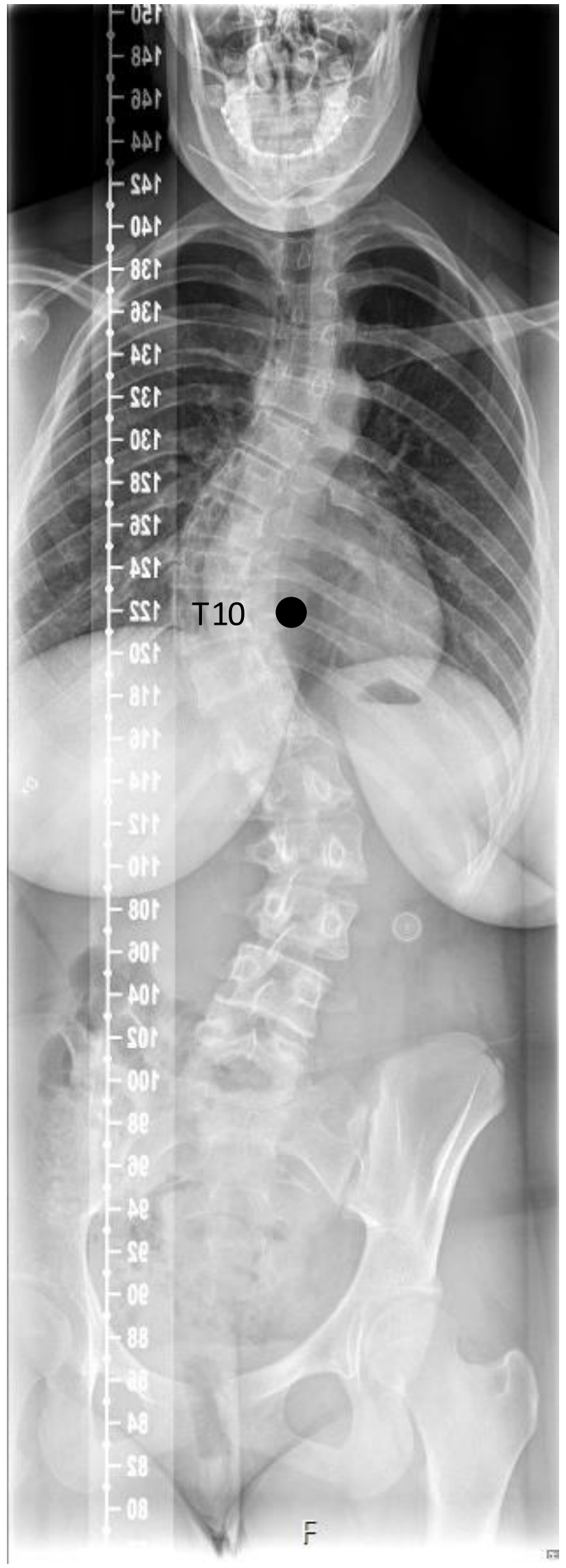


Patient 23

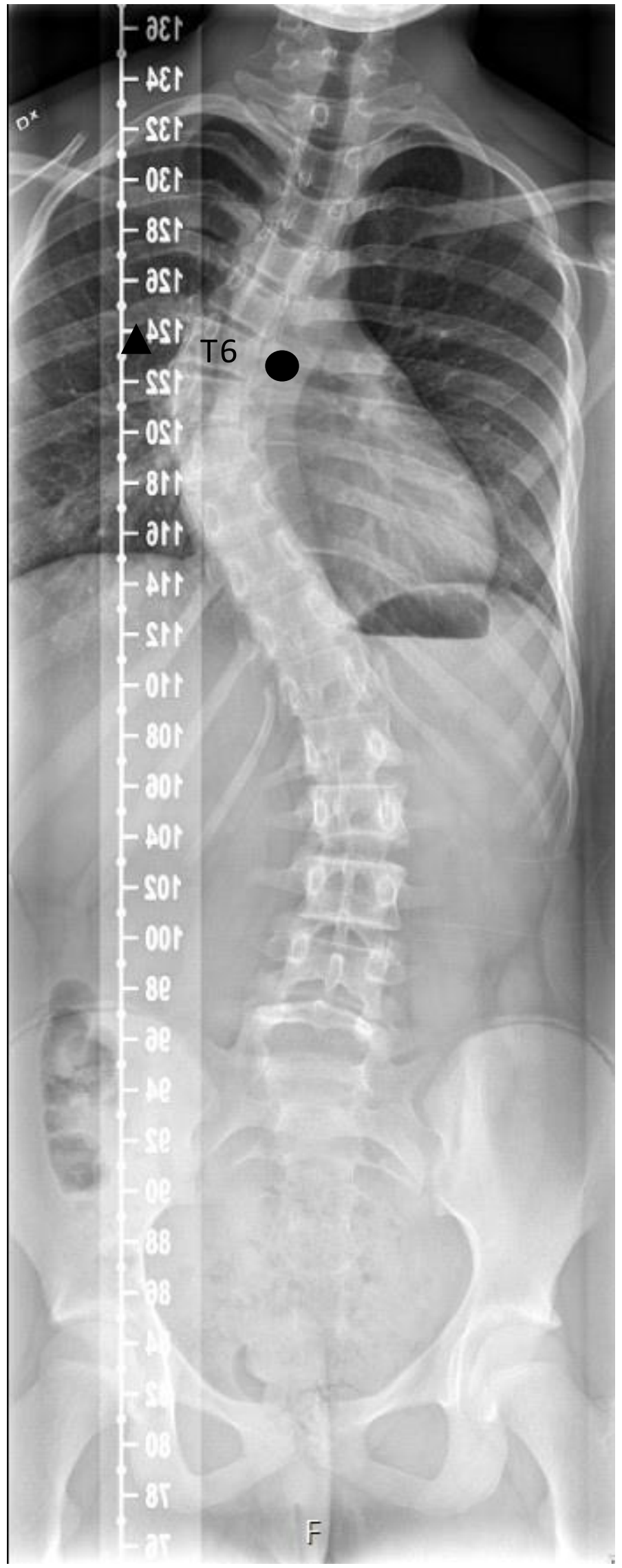


Patient 24

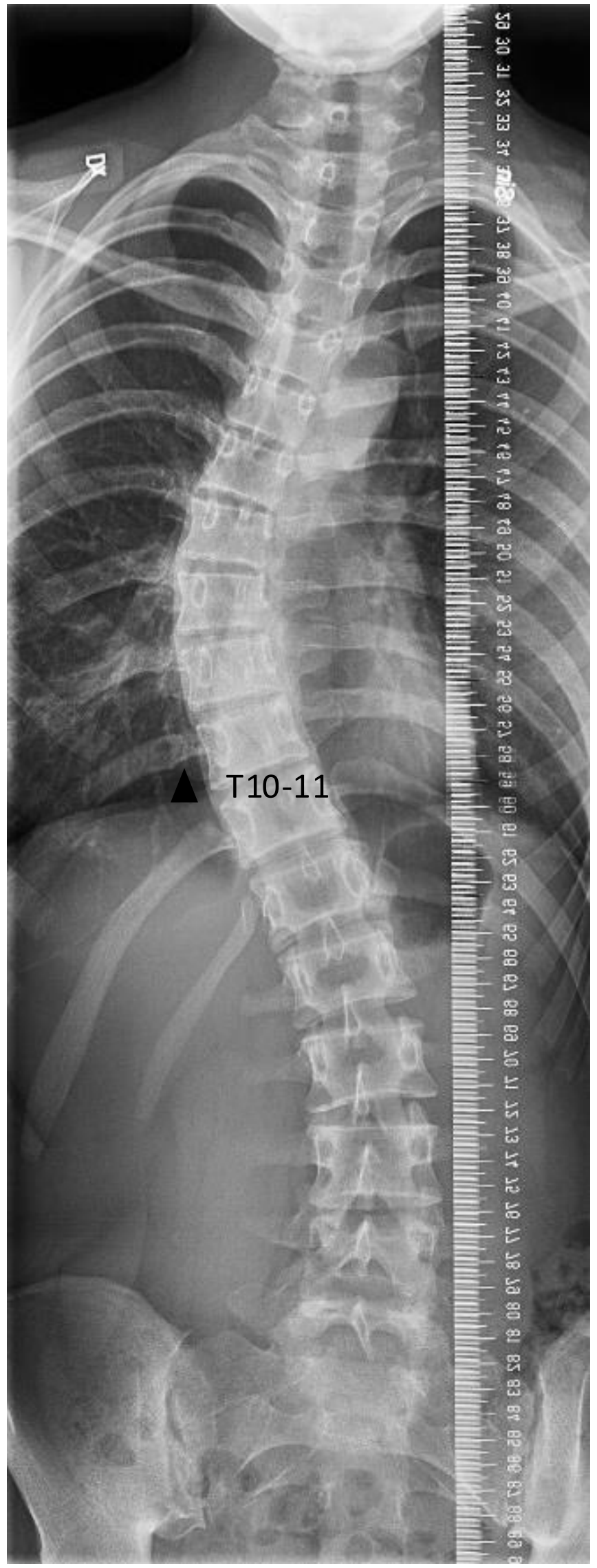

Patient 25

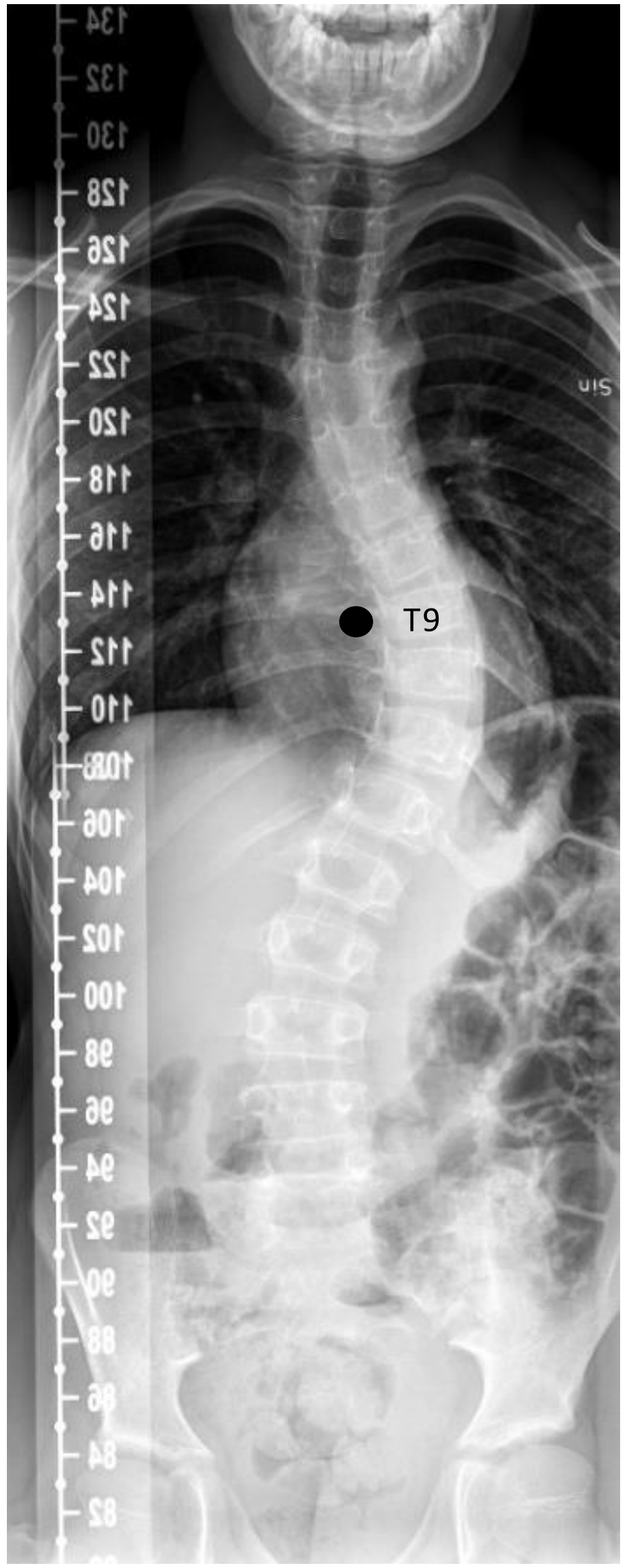


Patient 26

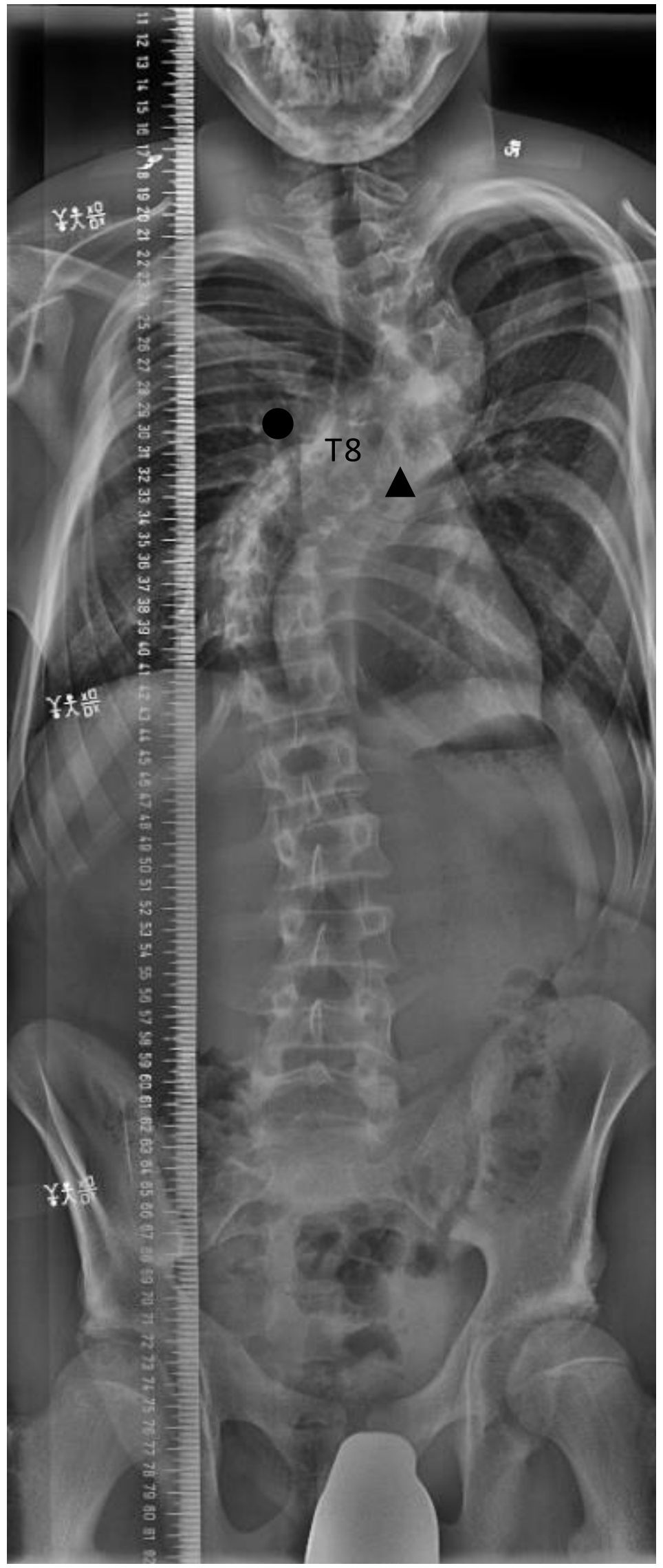


Patient 27

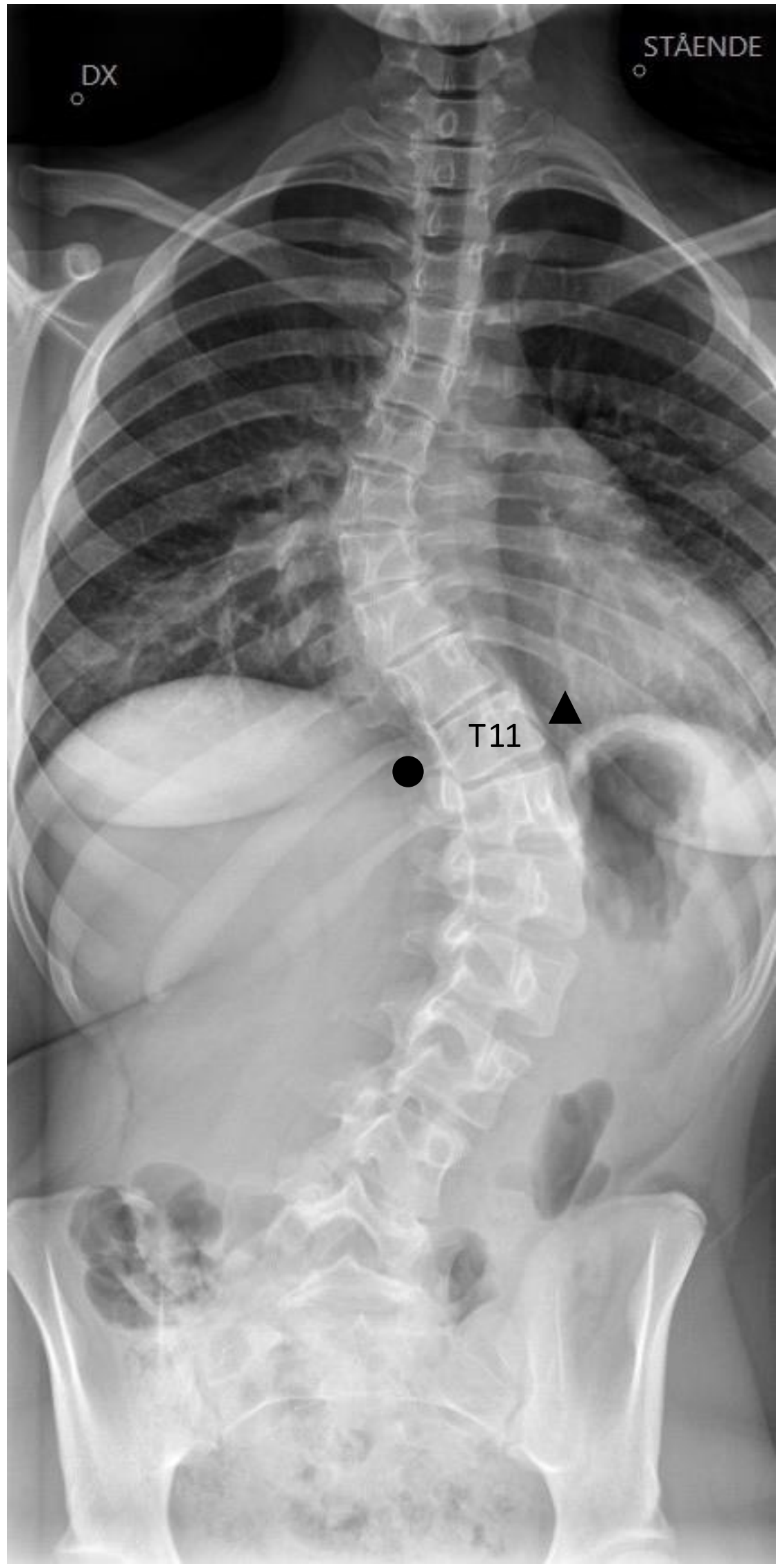


Patient 28

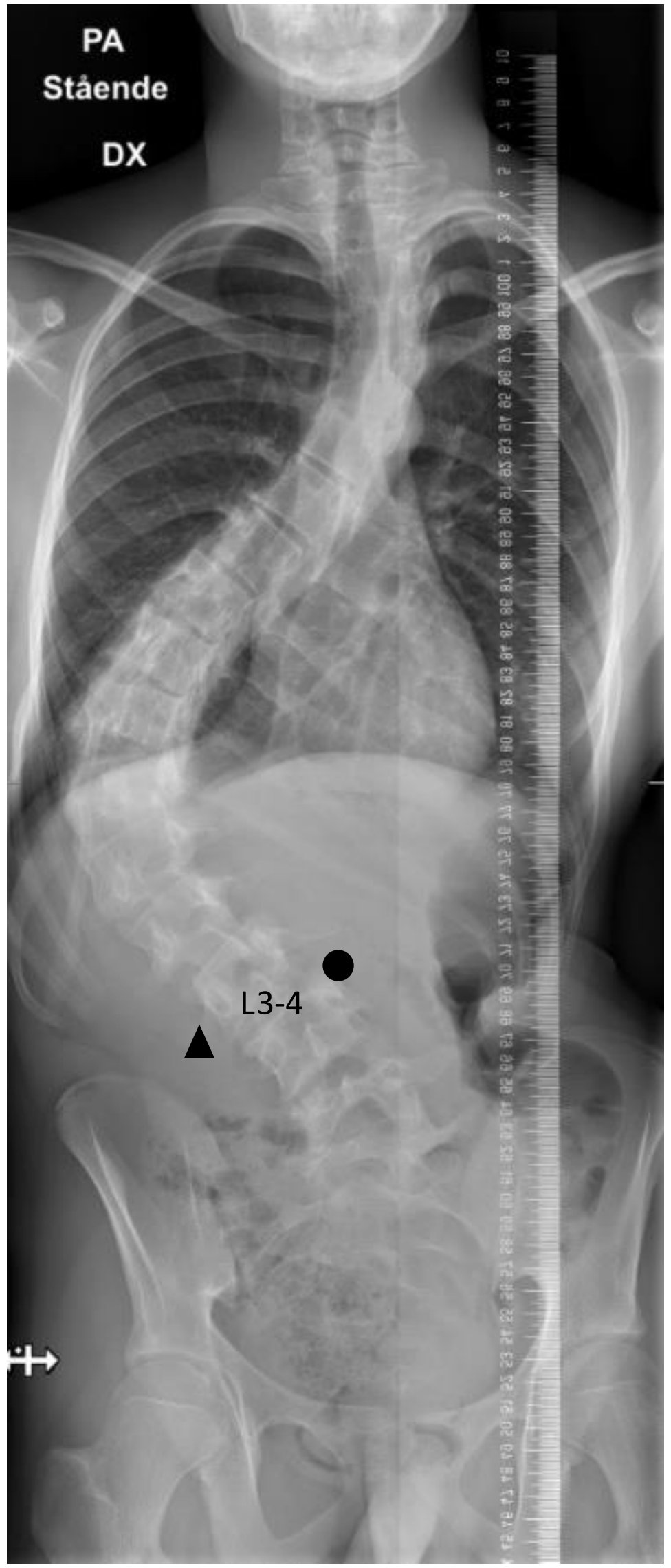

Patient 29

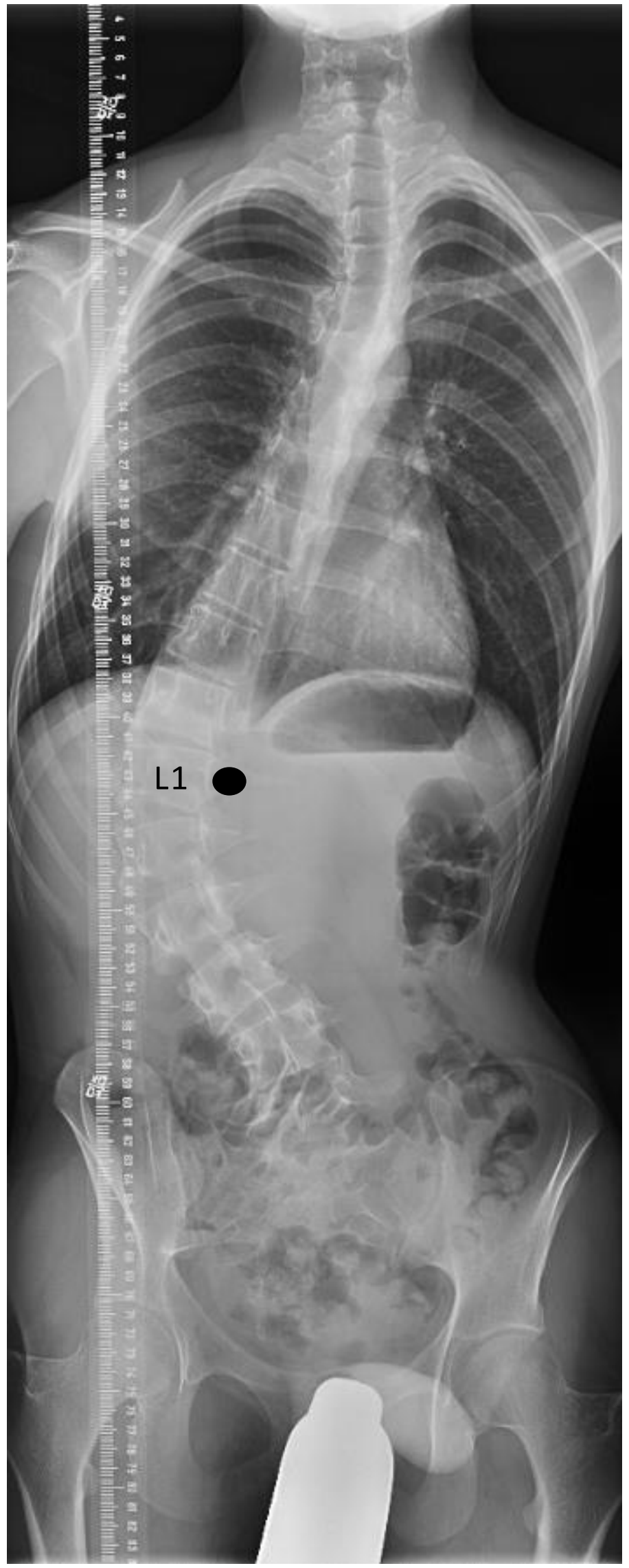


Patient 30

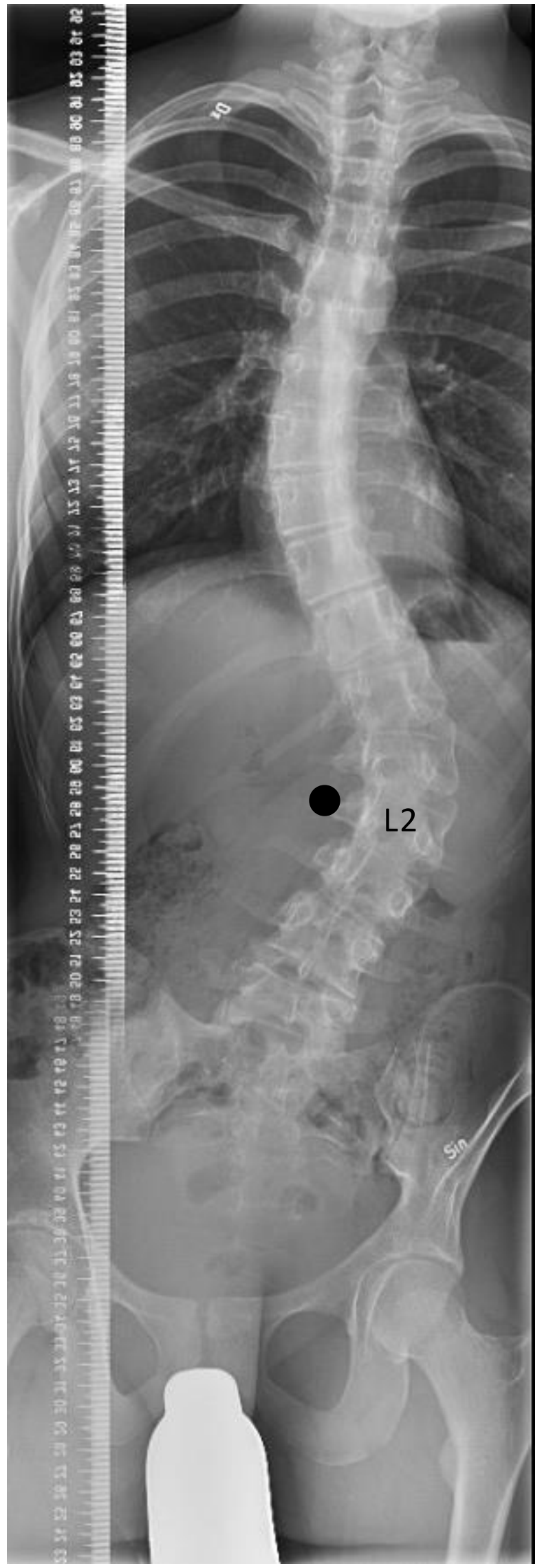


Patient 31

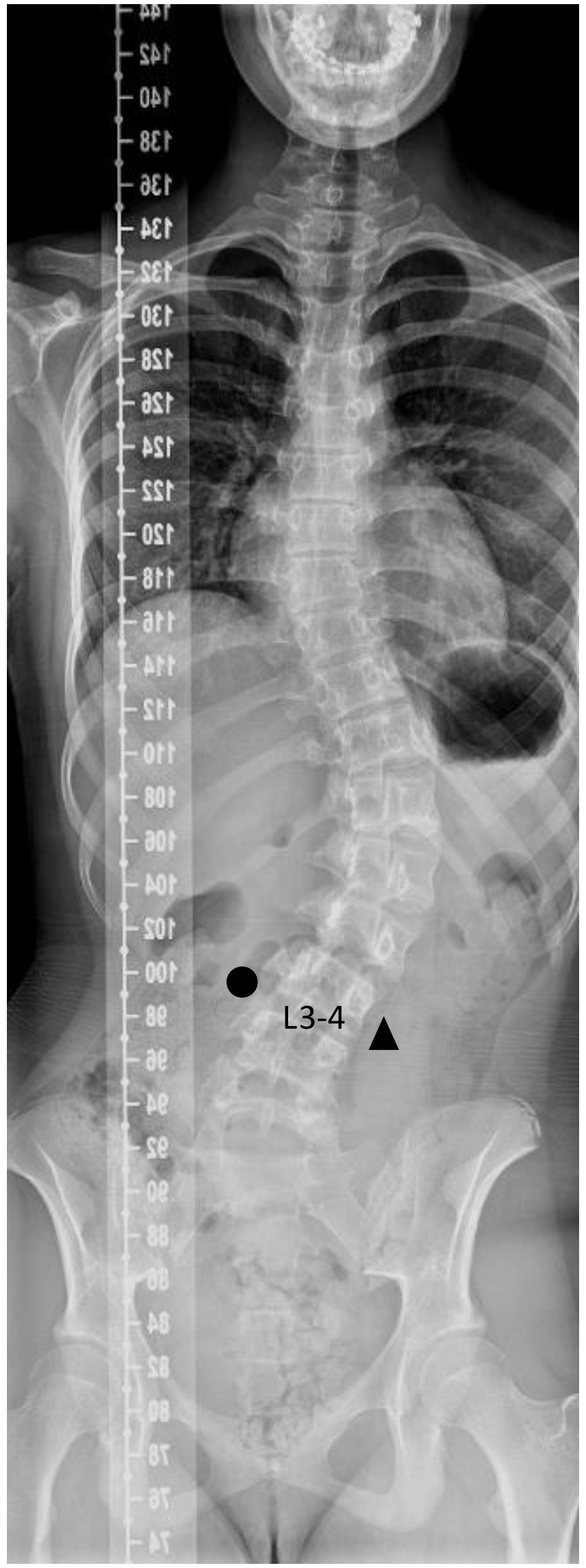


Patient 32

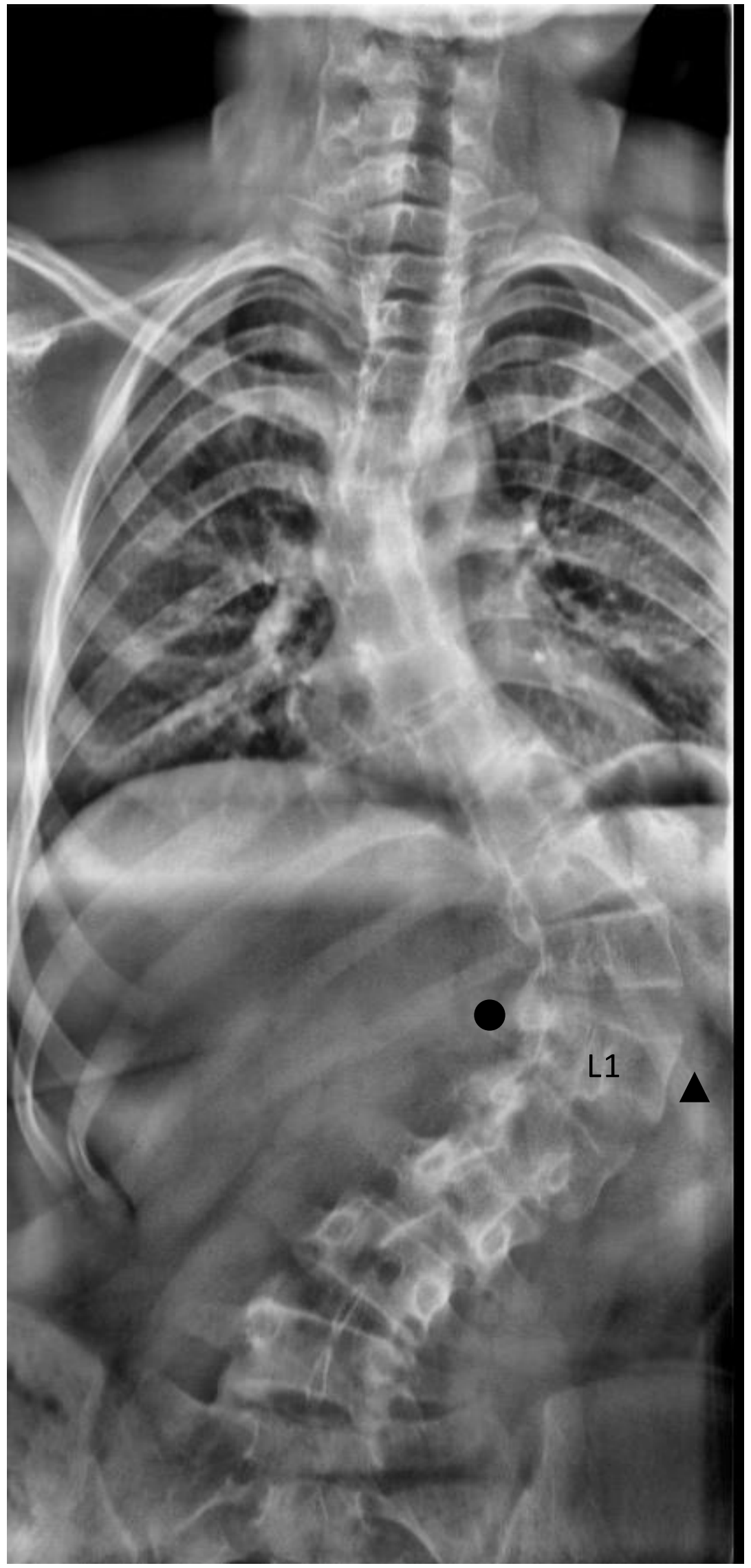

Patient 33

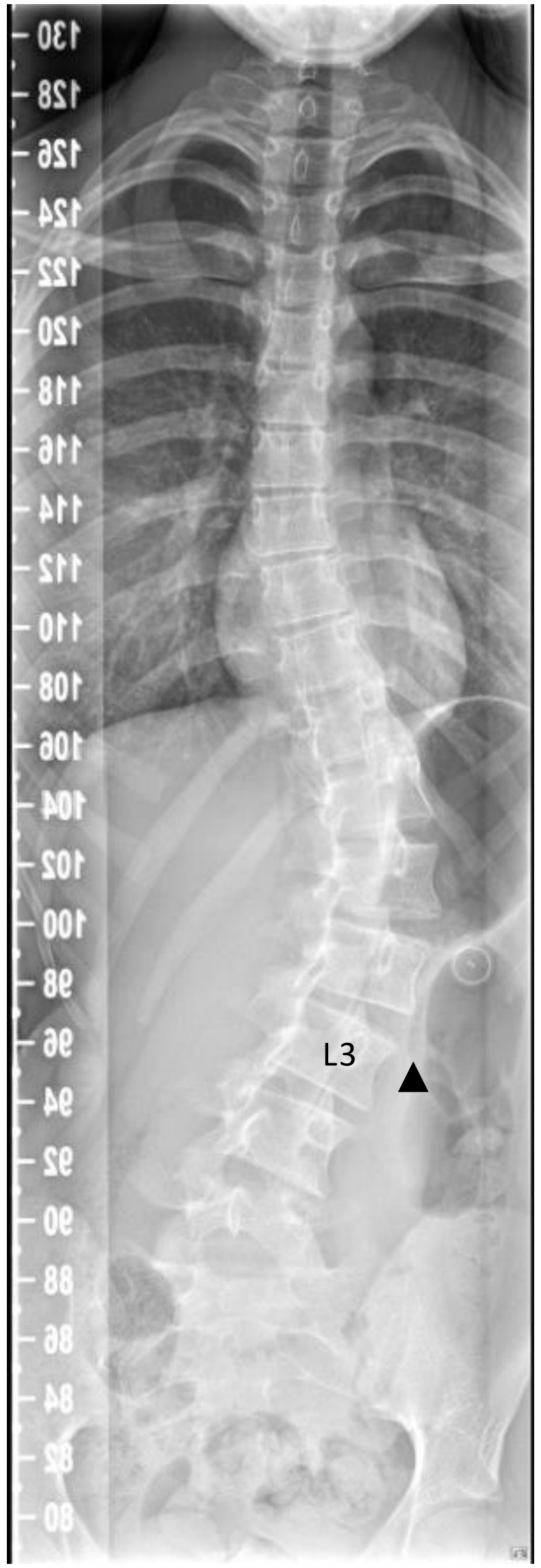


Patient 34

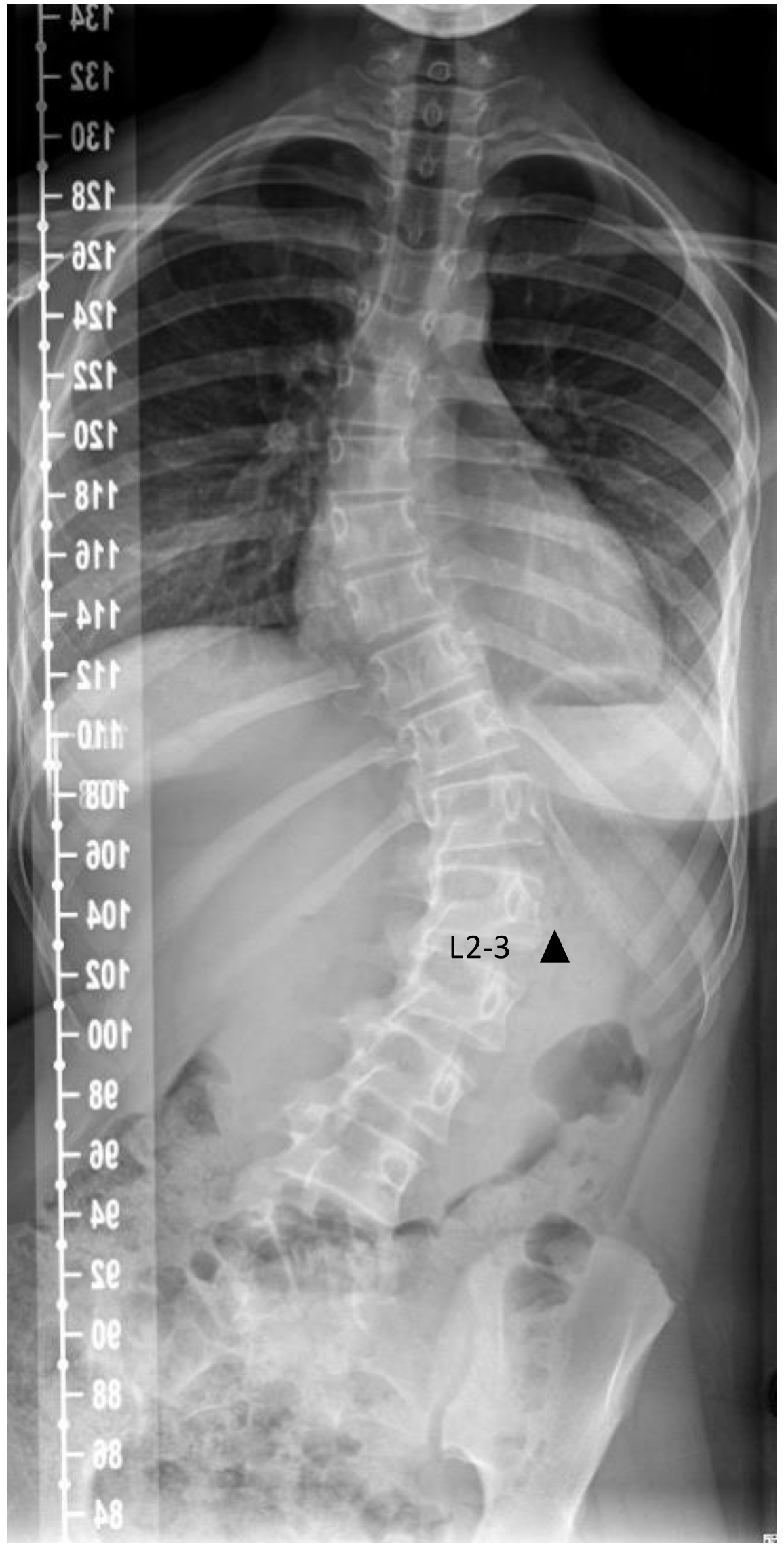


Patient 35

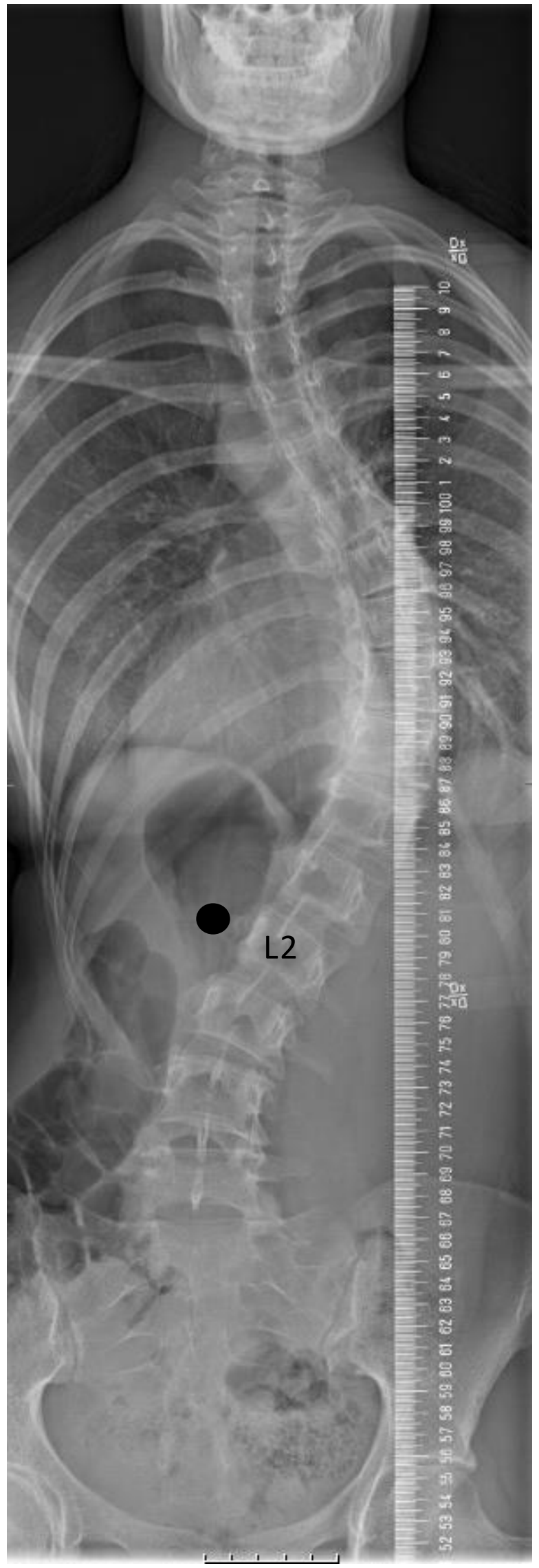

Control bilateral samples n=5

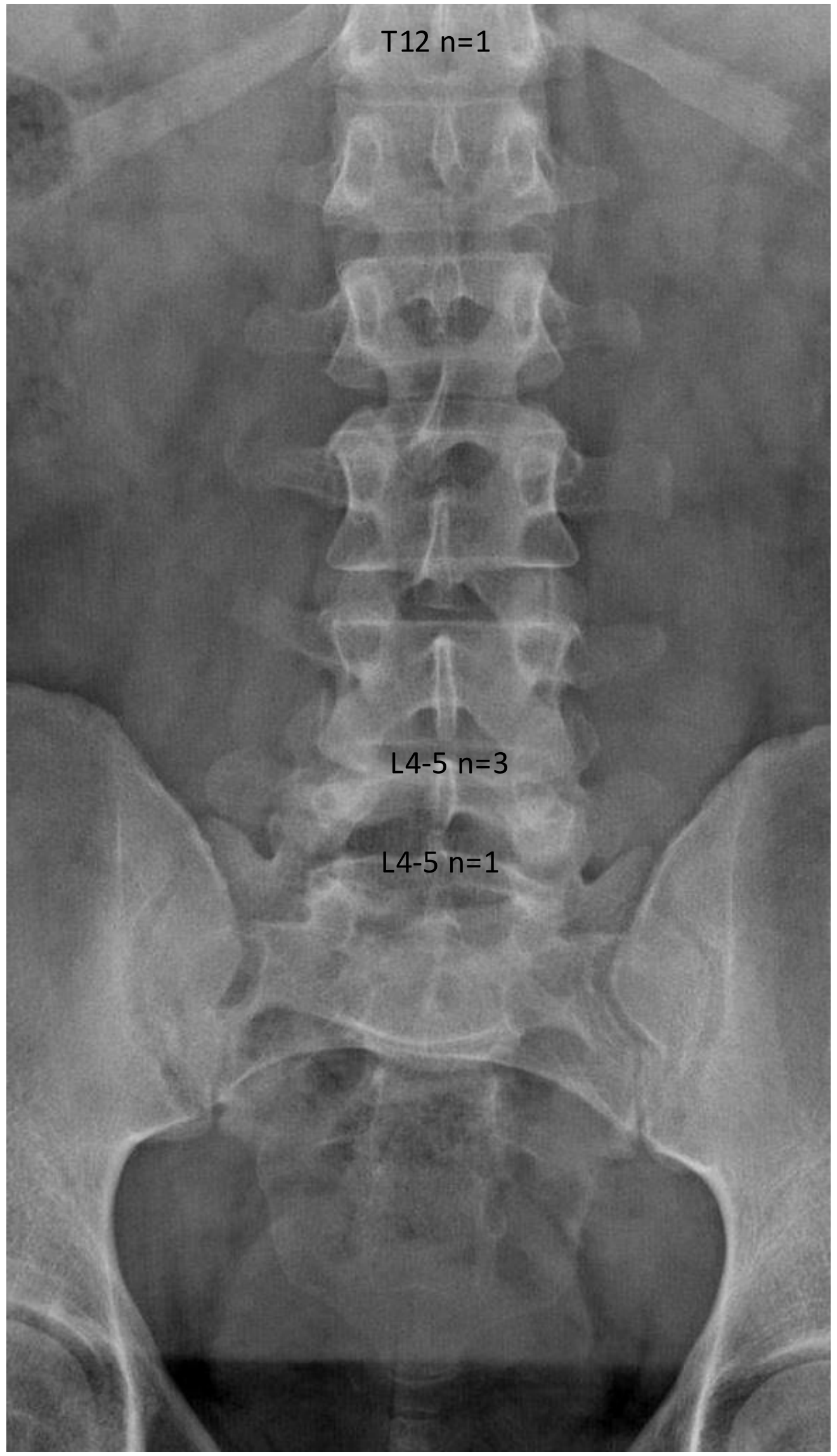


Control left side n=3

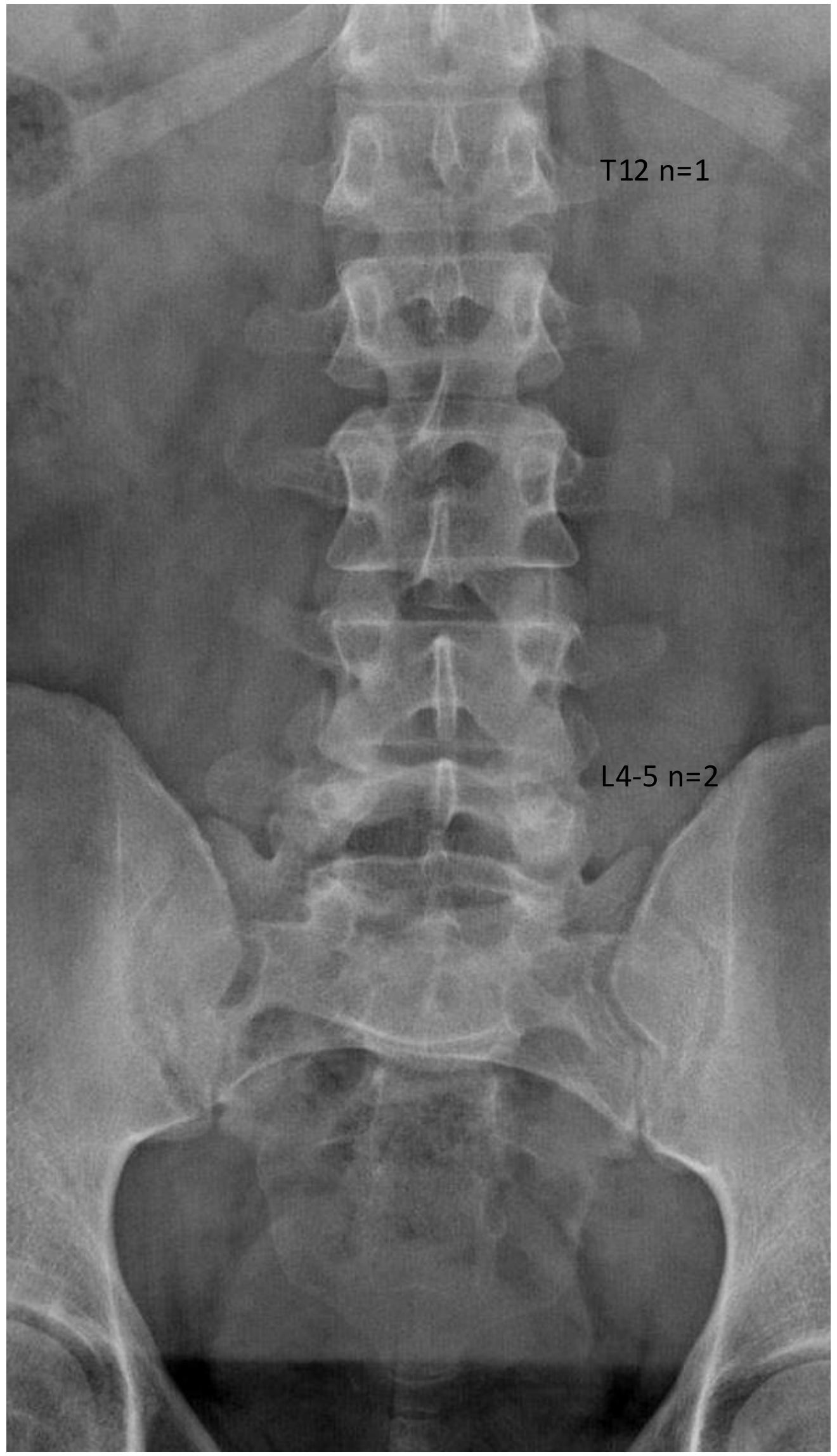


Control right side n=9

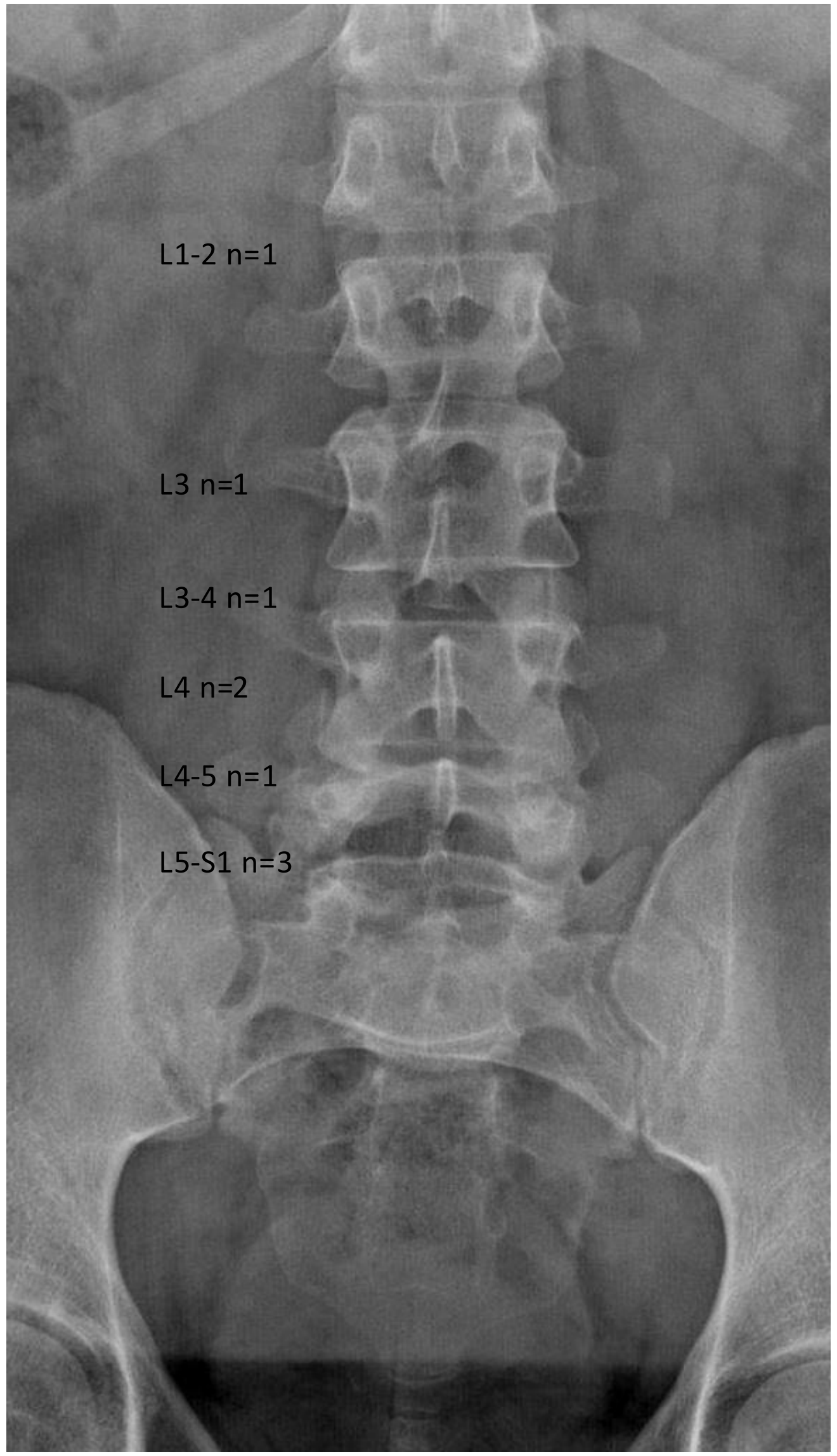

Supplement: Supplementary file 1 [file JSP2-9-e70214-s006.pdf]
