# Supplementary material for: Fetal Pathogenesis of Scoliosis Suggested by Asymmetry of Gene Expression in Paravertebral Muscles
Source: JOR Spine. 2026 Aug 16;9(3):e70214. doi: 10.1002/jsp2.70214 (PMC13478252; doi:10.1002/jsp2.70214)
Supplement: Supplementary file 2 [file JSP2-9-e70214-s008.pdf]

	Scoliosis Cases (n=35)	Controls (n=17)
**Sex n (%)**		
Females	24 (69)	5 (29)
Males	11 (31)	12 (70)
**Age at surgery m (SD)**	15.9 (2.4)	17.1 (2.2)
**Diagnosis (n)**		
Idiopathic scoliosis	35	0
Lumbar Disc herniation	0	11
Spondylolisthesis	0	2
Tumor	0	3
Scheuermann's kyphosis	0	1
**Major curve cobb angle m (SD)**	59 (10)	n/a
**Major curve type n (%)**		n/a
Thoracic	25 (71)	
Thoracolumbar	5 (14)	
Lumbar	5 (14)	
**Major curve convexity n (%)**		n/a
Right	24 (69)	
Left	11 (31)	
**Sampling level n (%)**		
T5/T6 & T6	2 (5.7)	0 (0)
T7/T8 & T8	2 (5.7)	0 (0)
T8/T9 & T9	3 (8.6)	0 (0)
T9/T10 & T10	5 (14.3)	0 (0)
T10/T11 & T11	4 (11.4)	0 (0)
T11/T12 & T12	6 (17.1)	2 (11.8)
T12/L1 & L1	3 (8.6)	0 (0)
L1/L2 & L2	3 (8.6)	1 (5.9)
L2/L3 & L3	4 (11.4)	1 (17.6)
L3/L4 & L4	3 (8.6)	3 (41.2)
L4/L5 & L5	0 (0)	7 (17.6)
L5/S1 & S1	0 (0)	3 (17.6)
